# Polymorphism-driven transcriptomic changes in anthelmintic metabolism pathways of *Anisakis simplex* s.s. L3 larvae

**DOI:** 10.1186/s13071-025-07197-w

**Published:** 2025-12-19

**Authors:** Mateusz Maździarz, Iwona Polak, Lukasz Paukszto, Monika Szczecińska, Elżbieta Łopieńska-Biernat

**Affiliations:** 1 Department of Botany and Evolutionary Ecology, Faculty of Biology and Biotechnology, University of Warmia and Mazury in Olsztyn, Plac Łódzki 1, 10-721 Olsztyn, Poland; 2 Department of Biochemistry, Faculty of Biology and Biotechnology, University of Warmia and Mazury in Olsztyn, Michała Oczapowskiego 2, 10-719 Olsztyn, Poland

## Abstract

**Background:**

Helminth infections continue to pose major challenges in human and veterinary medicine, with additional complications arising from the emergence of anthelmintic resistance. *Anisakis simplex* sensu stricto (*A. simplex* s.s.), a zoonotic nematode transmitted through the consumption of fish, is of growing concern due to its allergenic potential and clinical relevance. However, the molecular mechanisms underlying the parasite’s response to anthelmintic treatment remain poorly defined.

**Methods:**

Third-stage larvae (L3) of *A. simplex* s.s. were exposed to three widely used anthelmintics: albendazole (ALB), ivermectin (IVC) and pyrantel (PYR). High-throughput RNA sequencing was combined with differential gene expression, multivariate alternative splicing analysis (Replicate Multivariate Analysis of Transcript Splicing [rMATS] v3.2.5 computational tool) and single nucleotide variant (SNV) profiling with Oxford Nanopore sequencing. Drug-specific effects were assessed across protein-coding genes, long non-coding RNAs (lncRNAs) and splicing events.

**Results:**

Distinct transcriptomic features, including splicing and sequence variants, were observed across treatments, with ALB primarily altering the expression of cuticle-associated genes, IVC inducing extensive alternative splicing in immune-related pathways and PYR exposure linked to widespread SNVs in neuronal projection and metabolic genes. Significant splicing events included exon skipping in the trehalase gene (ALB) and combined skipped exon/alternative 5′ splice site events in moesin/ezrin/radixin-like protein 1 (IVC). A stop/splice-region SNV in trehalose phosphatase was detected with PYR exposure, highlighting coordinated disruption of the trehalose metabolism pathway. Across treatments, 68, 83 and 95 protein-coding genes with allelic variation were identified for ALB, PYR and IVC, respectively, including genes involved in detoxification, oxidative stress, cytoskeletal remodeling and transcriptional regulation.

**Conclusions:**

Our findings reveal complex, drug-specific regulatory mechanisms in *A. simplex*, encompassing transcriptional remodeling, alternative splicing and functional SNVs. Novel modulation of trehalose metabolism and cytoskeletal genes, alongside potential roles for ABC transporters and RNA-binding proteins, suggests diverse adaptive strategies underlying anthelmintic tolerance. This study represents the first integrated transcriptomic and variant-level analysis of *Anisakis* under drug pressure and provides new insights into molecular resistance mechanisms in marine nematodes, with implications for therapeutic innovation and monitoring strategies.

**Graphical Abstract:**

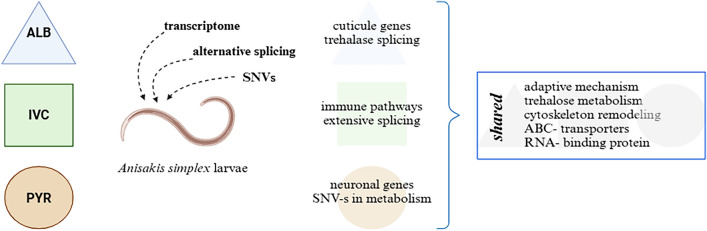

**Supplementary Information:**

The online version contains supplementary material available at 10.1186/s13071-025-07197-w.

## Background

Anthelmintics are the cornerstone of measures aimed at controlling parasitic nematodes affecting humans and animals. Three main groups of anthelmintic drugs, namely macrocyclic lactones (e.g. ivermectin [IVC]), imidothiazoles (e.g. pyrantel [PYR]) and benzimidazoles (e.g. albendazole [ALB]), are commonly used in therapeutic and preventive regimens. Each group targets distinct biological pathways: macrocyclic lactones act on glutamate- and γ-aminobutyric acid (GABA)-gated chloride channels, leading to neuromuscular paralysis; imidothiazoles depolarize muscle membranes by targeting cholinergic receptors; and benzimidazoles inhibit microtubule polymerization and disrupt energy metabolism via fumarate reductase inhibition [1–5]. Despite their broad-spectrum activity, the efficacy of these drugs varies across nematode species and developmental stages. Increasingly, reports of reduced efficacy and drug resistance have emerged from diverse parasitic taxa [6, 7]. Resistance can arise through multiple mechanisms, including point mutations in drug target genes, altered expression of detoxification enzymes (e.g. cytochrome P450 enzymes, glutathione S-transferases [GSTs]) and enhanced efflux via ABC transporters [8–12]. Moreover, resistance is often multifactorial, involving complex regulatory networks that remain poorly characterized in many parasites. Anthelmintics and their efficacy remain controversial and debated across the fields of parasitology and pharmacology. The absence of effective vaccines and limited access to adequate sanitation in many endemic regions hamper the ability to disrupt parasite life-cycles and control transmission [7]. The widespread and repeated use of anthelmintics increases selection pressure, raising the risk of multidrug resistance and treatment failure. Additionally, experiments using in vitro and in vivo models often require non-physiological drug dosages that fail to reflect true pharmacodynamics in parasitic organisms [13]. As a result, there is a growing consensus that next-generation antiparasitic compounds must be designed with species-specific or stage-specific selectivity, taking into account the ecological and biological context of the parasite within its host environment.

In this context, *Anisakis simplex *sensu stricto (*A. simplex* s.s.) represents a unique challenge. This marine nematode causes anisakiasis in humans following the consumption of raw or undercooked infected fish. Its third-stage (L3) larvae, which are infective to humans, can trigger acute gastrointestinal symptoms as well as allergic reactions [3–5, 14]. The increasing prevalence of *Anisakis* larvae in commercial fish, combined with the lack of effective treatments, underscores the urgency of understanding its biology and drug susceptibility [14, 15]. While most research on anthelmintic resistance has focused on terrestrial nematodes, marine parasites like *A. simplex* s.s. remain underexplored. Preliminary studies suggest that conventional drug targets may not be conserved in this species and that exposure to anthelmintics such as IVC or PYR results in transcriptional changes not fully explained by classical mechanisms [8, 16–18]. Results from proteomic and transcriptomic analyses indicate a potentially divergent response to drug pressure, including activation of stress-related proteins and redox pathways [19]. Elucidation of the molecular mechanisms underlying drug response in parasitic nematodes is facilitated by the application of high-throughput transcriptomic approaches that provide a powerful and comprehensive toolkit. Technologies such as RNA sequencing (RNA-Seq) and long-read sequencing enable in-depth analysis of gene expression, alternative splicing and sequence variation—including single nucleotide variants (SNVs)—within the parasite’s transcriptome. Comprehensive knowledge of these features is essential to understand the complex molecular adaptations to anthelmintic pressure [20, 21]. Recent evidence highlights the role of long non-coding RNAs (lncRNAs) and post-transcriptional regulatory mechanisms, such as alternative splicing, in modulating xenobiotic metabolism and stress responses [22]. LncRNAs can influence transcriptional activity, messenger RNA (mRNA) stability and translation efficiency, while alternative splicing may generate distinct protein isoforms with divergent or even opposing functions, potentially altering drug sensitivity or resistance [23, 24]. Together, these regulatory layers offer critical insights into the dynamic response of parasitic nematodes to anthelmintic exposure. In this study, we present an integrative transcriptomic analysis of *A. simplex* s.s. L3 larvae exposed to three common anthelmintics: ALB, IVC and PYR. Drug-induced changes in gene expression, lncRNA profiles, and alternative splicing were analyzed using short-read sequencing, while single-nucleotide variants (SNVs) were identified using long-read sequencing.. By combining these data, we aim to uncover molecular pathways and regulatory elements involved in drug response, with the long-term goal of informing future monitoring of anthelmintic resistance and the rational design of novel therapeutics for zoonotic nematodes.

## Methods

### *In vitro* culture of *A. simplex* s.s larvae

Third-stage (L3) larvae of *A. simplex* s. s were isolated from Baltic herring (*Clupea harengus membras*). Taxonomic identification of the larvae was performed using conventional real-time PCR targeting the internal transcribed spaced (ITS) region, employing the Anis Sensitive Sniper RT-PCR Kit (A&A Biotechnology, Gdańsk, Poland) [16]. The isolated larvae were first thoroughly washed in sterile 0.9% NaCl to remove any contaminants and then cultured in vitro for 12 h in RPMI-1640 medium in 6-well plates under standard conditions (37 °C, 5% CO_2_, and 95% relative humidity), following the method described by Iglesias et al. [25]. The experiment consisted of a control group (CTR; cultured without drugs but with 0.1% dimethyl sulfoxide [DMSO]) and three treatment groups, with larvae in each treatment group exposed to one of three anthelmintic compounds: IVC (10⁻⁷ M), PYR (10⁻⁶ M) and ALB (10⁻⁶ M). All drugs were dissolved in DMSO, and the final DMSO concentration in both the CTR and treatment groups was adjusted to 0.1% (v/v). The drug doses used in this research were chosen following preliminary studies [8, 16, 19], medicine distributor leaflets and literature review [26–31]. For each drug treatment group and the CTR group, four samples were prepared (10 larvae per sample) and all larvae remained alive throughout the experiment. At the end of the culture period, all larvae were collected and stored at − 80 °C until transcriptome analysis.

### Preparation of samples and sequencing

Transcriptomic analysis of total RNA isolated from L3 larvae of *A. simplex* s.s cultivated with and without drug treatments was performed using TRIzol reagent. (Cat. No. 15596026; Invitrogen, Thermo Fisher Scientific, Waltham, MA, USA) together with the PureLink RNA Mini Kit (Cat. No. 12183018 A; Invitrogen, Thermo Fisher Scientific) according to the manufacturer’s instructions. RNA quantity and integrity were checked using the Bioanalyzer 2100 and the RNA 6000 Nano LabChip Kit (Cat. No. 5067–1511; Agilent Technologies, Santa Clara, CA, USA). Only high-quality RNA samples with RNA Integrity Number (RIN) > 7.0 were used to generate the sequencing library (L3 CTR group, 4 samples; L3 ALB treatment group, 4 samples; L3 IVC treatment group, 4 samples; L3 PYR treatment group, 4 samples; in total, 16 samples). Approximately 5 µg of total RNA was used to remove ribosomal RNAs using the Ribo-Zero Gold rRNA Removal Kit (Cat. No. MRZG12324; Illumina Inc., San Diego, CA, USA) according to the manufacturer’s instructions. After removal of the ribosomal RNAs, the remaining RNAs were fragmented (Cat. No. E6150S; NEBNext® Magnesium RNA Fragmentation Module, New England Biolabs, Ipswich, MA, USA) into short fragments using divalent cations at a high temperature (94 ℃, 5–7 min). Subsequently, the cleaved RNA fragments were reverse transcribed with SuperScriptTM II Reverse Transcriptase (Cat. No. 1896649; Invitrogen, Thermo Fisher Scientific) to generate complementary DNA (cDNA), which was then used to synthesize U-labeled, second-stranded DNAs with* Escherichia coli* DNA polymerase I (Cat No. m0209; New England Biolabs), RNase H (Cat. No. m0297; New England Biolabs) and dUTP solution (Cat. No. R0133; Thermo Fisher Scientific). An A-base was then added to the blunt ends of each strand to prepare them for ligation with the indicated adapters. Each adapter contained a T-base overhang for ligation of the adapter to the fragmented A-tailed DNA. The dual-index adapters were ligated to the fragments, and size selection (300–600 bp) was performed using AMPureXP beads (Cat. No. A63881; Beckman Coulter Life Sciences, Indianapolis, IN, USA). After treatment of the U-labeled secondary-stranded DNA with the heat-labile UDG enzyme (Cat No. m0280; New England Biolabs), the ligated products were amplified by PCR under the following conditions: an initial denaturation at 95 °C for 3 min; 8 cycles of denaturation at 98 °C for 15 s, annealing at 60 °C for 15 s and extension at 72 °C for 30 s; and then a final extension at 72 °C for 5 min. The average insert size of the final cDNA libraries was 300 ± 50 bp. Finally, we performed 2 × 150-bp paired-end sequencing (PE150) on an Illumina Novaseq™ 6000 (LC BioTechnology CO., Ltd., Hangzhou, China) according to the manufacturer’s recommended protocol.

All bioinformatics analyses (RNA-Seq preprocessing, transcriptome assembly, differential expression, alternative splicing detection and variant calling) were performed using standardized command-line workflows. A complete list of commands, parameters, and options used is provided in Additional file 2: Table S1.

### Expression profiling

The quality of the sequencing was assessed using FastQC software (https://www.bioinformatics.babraham.ac.uk/projects/fastqc; accessed 4 April 2024). After RNA-Seq, Illumina adaptors and poly-A segments were excised using the Trimmomatic v.0.39 trimming tool with the following parameters: CROP:140 LEADING:20 TRAILING:20 MINLEN:140 AVGQUAL:20 (https://www.usadellab.org/cms/? page = trimmomatic) [32]. Reads shorter than 120 nucleotides and with an average Phred quality score < 20 were excluded from the dataset. High-quality FASTQ reads were mapped to the *A. simplex* PRJEB496 genome reference from WormBase ParaSite using STAR v.2.7.11.a. with the following command parameters: - -outSAMmapqUnique 50 - -outFilterMultimapNmax 20 - -outFilterMismatchNmax 999 - -outFilterMismatchNoverLmax 0.04 - -alignSJoverhangMin 8 - -alignSJDBoverhangMin 1 - -alignIntronMin 20 - -alignIntronMax 1000000 - -alignMatesGapMax 1000000 [33]. The resulting binary alignment map (BAM) files were then used to create annotations with StringTie v2.2.1 [34]. The Ballgown v.2.36 library [35] was then used to quantify expression and perform differential expression analysis between the CTR versus ALB treatment group, CTR versus IVC treatment group and CTR versus PYR treatment group. The genes with low expression were excluded from the study following read counting during the R scripting procedure. The gene-level FPKMs for each sample were estimated using the Ballgown tool. The RNAs with log2FoldChange (log2FC) > 1 and an adjusted* p*-value (*p*-adj) < 0.05 were considered to be statistically significant. Subsequently, the identified transcripts were categorized into three groups: (i) differentially expressed genes (DEGs); (ii) differentially expressed long non-coding RNAs (DELs); and (iii) other RNAs. The gene descriptions were assigned using the biomaRt v.2.60.1 [36] package and ENSEMBL annotations [37]. Pearson correlations between the FPKM values for DEGs and DELs were determined using the *cor* function from the stats v.4.4.1 package in the R environment. The absolute threshold values for this coefficient (Pearson Correlation Coefficient) were > 0.7.

### Real-time PCR

The mRNA level of randomly selected DEGs was determined by real-time PCR according to the general approach described by Polak et al. [16] with modifications. Total RNA was extracted as described in section Preparation of samples and sequencing using the TRIzol reagent in combination with the PureLink RNA Mini Kit (Cat. No. 12183018 A; Invitrogen, Thermo Fisher Scientific) according to the manufacturer’s guidelines. cDNA synthesis was performed using the Invitrogen SuperScript® VILO™ cDNA Synthesis Kit (Cat. No. 11754; Thermo Fisher Scientific Baltics, Vilnius, Lithuania) according to the protocol provided. Specific primers were designed using Primer3Plus software based on sequences from GenBank (Additional file 2: Table S2). The real-time quantitative PCR (qPCR) reactions were prepared using Applied Biosystems™ PowerUp™ SYBR™ Green Master Mix (Cat. No. A25780; Thermo Fisher Scientific Baltics) containing 5 μl of 2× Master Mix, 500 nM of each primer, 10 ng of cDNA and nuclease-free water to a final volume of 10 μl. Amplifications were carried out on a QuantStudio™ 3 Real-Time PCR System (Applied Biosystems™, Thermo Fisher Scientific) in four technical replicates. The reaction conditions were: an initial denaturation at 95 °C for 10 min; followed by 40 cycles of 15 s at 95 °C, 60 s at 60 °C, and 30 s at 72 °C. Reaction specificity was verified by melting curve analysis. The relative mRNA concentrations were quantified using the comparative Pfaffl method [38]. Changes were plotted against the untreated control and normalized against endogenous reference genes: actin (KP200883) and glyceraldehyde-3-phosphate dehydrogenase (KM496565) (relative quantification [RQ] = 1). Results were expressed as means ± standard deviations (SD) and plotted on a log₁₀ scale. Statistical significance for differences in expression of each of the 16 genes was assessed individually using ordinary one-way analysis of variance (ANOVA), followed by Dunnett’s multiple comparison test (comparing each treatment group to CTR) in Prism 10 (GraphPad Software Inc., San Diego, CA, USA), with *p*-values interpreted as follows: 0.0332 (*), 0.0021 (**), 0.0002 (***) and < 0.0001 (****).

### Alternative splicing events and differential analysis

The Replicate Multivariate Analysis of Transcript Splicing [rMATS] v3.2.5 computation tool (rMATS v.3.2.5 with options -t paired - -readLength 140 - -cstat 0.05) was used to discover differences alternative splicing events (ASEs) based on RNA-Seq raw reads mapped to the reference *A. simplex* genome [39]. The percent of splicing inclusion (PSI) values was calculated for all alternative splicing events (ASes) according to the reads aligned to the spliced junction sites. The differential alternative splicing events (DASes) between the used medicaments and CTR were statistically tested (false discovery rate [FDR] < 0.05). Moreover, DASes were filtered according to the differential level of the absolute (ΔPSI) > 0.1. The DASes were classified into five subtypes: alternative 5′ splice site (A5SS), alternative 3′ splice site (A3SS), mutually exclusive exons (MXE), retained intron (RI) and skipped exon (SE).

### Detection of single nucleotide variants and differential analysis

SNVs were also examined according to allele frequency differences between the CTR and three experimental treatments. In the first step of the SNV analysis, the mapping results and annotation files were used to carry out variant calling analysis by the bcftools v.1.22 mpileup function [40]. Low-quality variants were filtered out. Allele frequencies were compared directly between the three treatments and CTR using the Weir and Cockerham Fst index [41] as a measure of differentiation. All substitutions with Fst > 0.66 were qualified as significant. The converted VCF file and significant results were intersected using bedtools v.2.30 [42]. Binary alignment map (BAM) files were scanned by Rsamtools v.2.20 software [43] to obtain allele frequency for all significant SNVs. The procedure was repeated for the three anthelmintic drugs (ALB, PYR, IVC). The significant variants were annotated by SNPeff v.4.5 software [44] and further manually selected to the validation procedure. The selection process included: ordering by decreasing coverage in the range of the SNV; repeatability inside the condition; and finally extraction of sequences with a length of 1001 bp (two 500-bp flanking regions and a 1-bp target substitution).

### Gene ontology

Information about gene ontology (GO) was obtained through the eggNOG database [45]. Gene ontologies were searched using coding DNA sequences (CDS) from the *A. simplex* transcriptome. All DEGs, DASes and SNVs were scanned in this way. Genes were assigned to ontologies when the following criteria were met: Min. hit e-value 0.001, Min. hit bit-score 60, Min. % identity 40, Min. % query coverage 20 and Min. % subject coverage 20 [46].

### Visualization

The results were visualized using ggplot2 v.3.3.5 [47], circlize v.0.4.15 [48] and circos.ca v.0.69–8 [49] software packages. SNVs were visualized using Geneious Prime software version 2025.0.2 (Biomatters, Auckland, New Zealand). The Sankey diagram was visualized using the ggalluvial library v.0.12.5 [44].

### Confirmation SNV Oxford Nanopore sequencing

Total DNA was extracted using the Pure™ Cell & Tissue Micro Kit (Cat. No. 090–50; A&A Biotechnology) and quantified prior to amplification. Briefly, 58 single nucleotide polymorphism (SNP) loci were targeted, of which 21 were confirmed in PYR-treated samples, 16 in IVC-treated samples and 21 in ALB-treated samples. Primers were designed to amplify regions containing selected variants using the Primer3Plus web-interface. Confirmatory amplicons were obtained for 31 loci (10 PYR, 10 IVC, 11 ALB); all locus-specific primer sequences for CTR samples are listed in Additional file 2: Table S11. In each PCR reaction volume (25 μl), 4 ng of DNA template and 10 µM of each primer were used in the PCR reactions with GoTaq® G2 Hot Start Master Mix (M7823; Promega, Madison, WI, USA). Cycling conditions consisted of an initial cycle of 94 °C for 2 min; followed by 35 cycles of 94 °C for 15 s, 60 °C for 15 s, 68 °C for 30 s; with a final extension at 68 °C for 1 min. PCR products were purified using the PureLink® PCR Purification Kit (K3100; Invitrogen, Thermo Fisher Scientific) and verified by capillary electrophoresis on the QIAxel system (Qiagen, Hilden, German) with 15 bp/3 kb and 15 bp/2.5 kb markers. Sequencing libraries were prepared using the Oxford Nanopore Native Barcoding Kit 96 V14 (SQK-NBD114.96) following the manufacturer’s two-step ligation protocol (Oxford Nanopore Technologies plc, Oxford, UK): end-preparation, native barcode ligation, adapter ligation and clean-up with. Barcoded libraries were pooled, loaded onto an R9.4.1 flow cell (FLO-MIN106), and sequenced on a MinION Mk1C with MinKNOW v21.03.24 (all Oxford Nanopore Technologies). Raw signal data were base-called with Dorado v0.8 (https://github.com/nanoporetech/dorado), and reads were converted to FASTQ. Filtlong v0.2.1 was used to filter for high-quality reads, which were then mapped to the *A. simplex* reference genome using minimap2 (v2.30; -ax map-ont). Allele frequencies at polymorphic sites were calculated in Geneious Prime v2025.1.1 using the Find Variation/SNP function. This validation confirmed the presence of RNA-derived polymorphic sites within genomic DNA sequences.

## Results

### Comparative analysis of drug-induced transcriptomic alterations

**Fig. 1:**
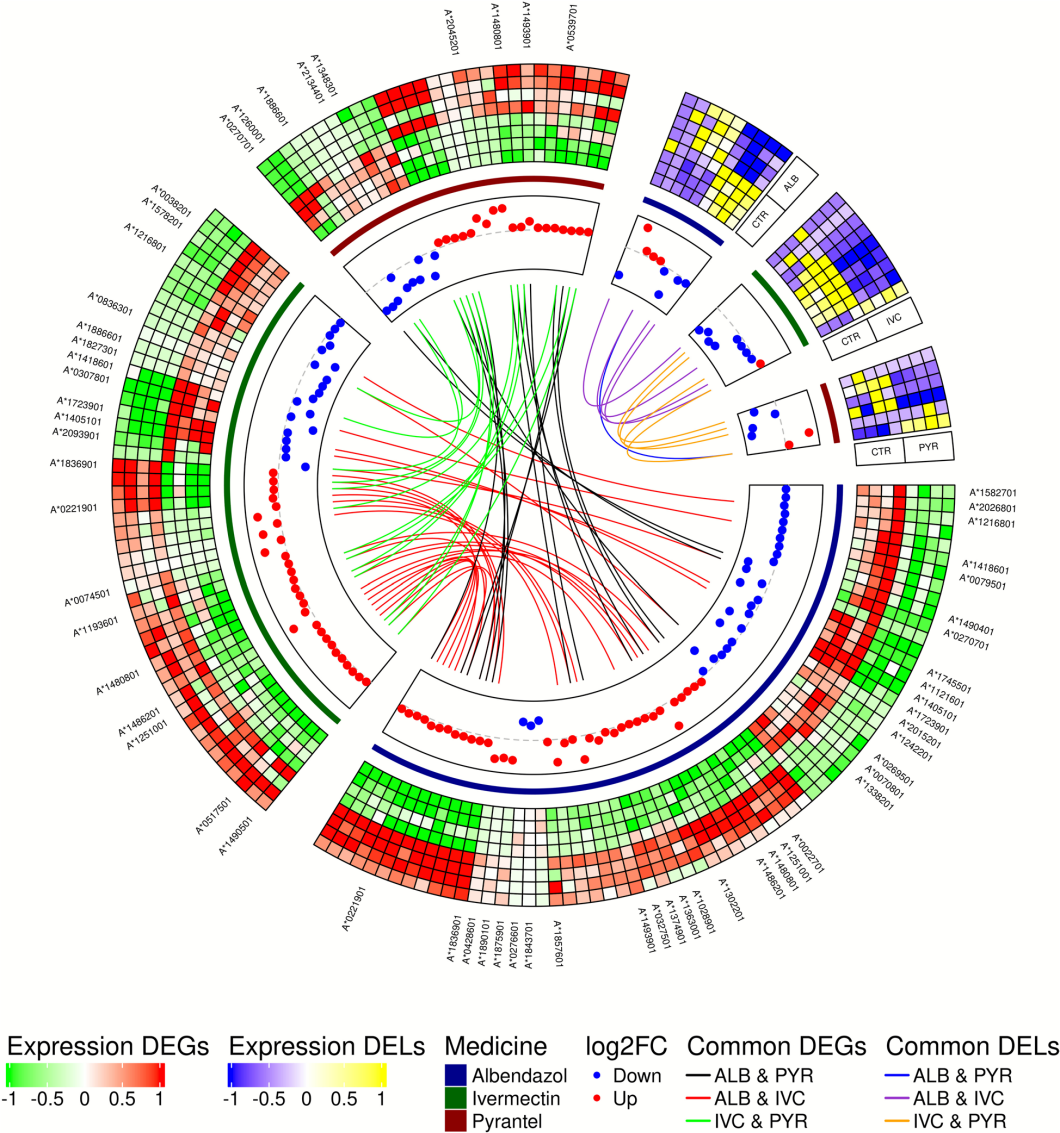
Comparison of DEG and DEL expression in response to drugs. The Circos chart shows a comparison of DEGs and DELs. The outermost track, or heatmap, displays *z*-scored expression values. The second track, a line graph, represents drug comparisons, with blue for ALB, green for IVC and red for PYR. The next track is a scatter plot showing log2FC values, where red dots indicate upregulated genes and blue dots indicate downregulated genes. The center of the chart features links that highlight the shared components: black for ALB and PYR DEGs, red for ALB and IVC DEGs, green for IVC and PYR DEGs, blue for ALB and PYR DELs, purple for ALB and PYR DELs and orange for IVC and PYR DELs. ALB, Albendazole; DEGs, differentially expressed genes; DELs, differentially expressed long-non-coding genes; IVC, ivermectin; PYR, pyrantel

The sequences obtained in the RNA-Seq experiment were characterized by very high quality, as evidenced by the following parameters. The average number of reads after filtering (Valid Data) was between 269 and 449 million reads per sample, representing over 97% of the raw reads (Raw Data) classified as valid (Valid Ratio ~ 21–31%). Base quality at the Q20 and Q30 levels was 99.9% and 97.7–97.8%, respectively, indicating an exceptionally low error rate (< 0.1% at Q20, < 0.03% at Q30) (Additional file 2: Table S3). A total of 65, 49 and 29 DEGs in the ALB, IVC and PYR samples, respectively, as well as eight, nine and six DELs in the ALB, IVC and PYR samples, respectively, were identified (Fig. 1; Additional file 2: Table S4). ALB samples contained 29 downregulated and 36 upregulated DEGs, IVC samples contained 20 downregulated and 29 upregulated DEGs and PYR samples contained 10 downregulated and 19 upregulated DEGs. PYR samples showed four downregulated and two upregulated DELs, ALB samples had five downregulated and four upregulated DELs and IVC samples exhibited eight downregulated and one upregulated DEL (Fig. 1; Additional file 2: Table S5). There were 12 common DEGs identified across all three treatments; additionally, 13 DEGs were shared between the PYR and IVC treatment groups, 26 were shared between the IVC and ALB treatment groups and 15 were shared between the ALB and PYR treatment groups (Fig. 1; Additional file 1: Figure S1; Additional file 2: Table S3). Additionally, four common DELs were identified for the PYR and IVC treatment groups, four were identified for the IVC and ALB treatment groups and one was identified for the ALB and PYR treatment groups. The MSTRG.1471.1 transcript DEL was common across all comparisons (Fig. 1; Additional file 1: Figure S2; Additional file 2: Table S4). The analysis of the interdependence between DEL and DEG transcripts showed important positive correlations, indicating a possible regulatory role of lncRNA (Pearson correlation > 0.7). In the ALB group, the strongest correlation (*r* = 0.76) was observed between the DEL MSTRG.1471 and the DEG encoding the* protein AT04676p*, which was identified on the basis of orthology to *Drosophila melanogaster*. The second strong link was the MSTRG.1342–mRNA* O-acyltransferase pair* (Additional file 2: Table S6). In the case of IVC, the DELs MSTRG.4373 and MSTRG.886 correlated strongly with the mRNA encoding* 1,2-dihydroxy-3-chain-5-methylthene dioxygenase*, while MSTRG.1095 and MSTRG.5177 correlated with a transcript encoding a protein with the *domain BPI2* (Additional file 2: Table S7). In response to PYR, DEL MSTRG.886 correlated strongly with *mRNA metalloendopeptidase *(Additional file 2: Table S8). These results indicate potential drug-specific transcriptional interactions between non-coding and coding transcripts. Notably, several genes with putative functions in cuticle formation, oxidative stress response and xenobiotic metabolism exhibited marked changes in expression. For example, a gene annotated as *Col_cuticle_N domain-containing protein* showed a substantial increase in expression (log2FC = 1.14), suggesting a possible role in modifying cuticular structure or permeability as an adaptive response to the three drugs. Conversely, under ALB treatment some *peroxidasin-like* transcripts were downregulated, potentially reflecting a suppressed oxidative defense mechanism (Additional file 2: Table S6).

Results from the RNA-Seq approach were confirmed using real-time PCR, which was used to demonstrate the expression of 16 randomly selected RNAs. Molecules for the validation were selected according to their functionality, expression levels and distribution across samples. There was a strong agreement (Pearson’s *r* = 0.85, *p*-value = 0.0039) between the RNA expression data obtained by real-time PCR and the RNA-Seq results (Additional file 1 Figures S3, S4; Additional file 2: Table S2).

### Differential alternative splicing under anthelmintic exposure

**Fig. 2:**
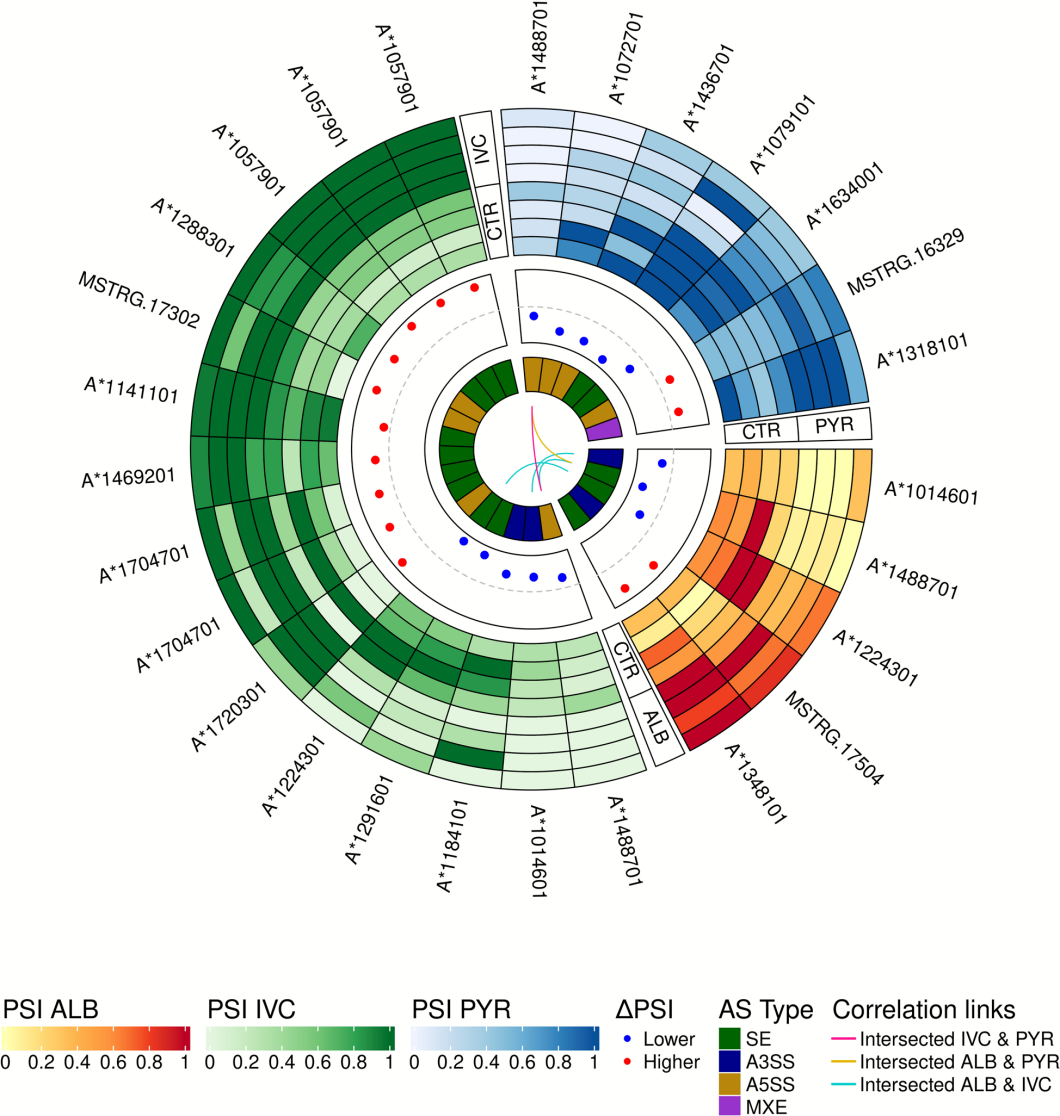
Circular heatmaps illustrate the associations between significant splicing events across three comparisons: IVC, ALB and PYR treatment groups. The heatmaps display the percentage of inclusion for each comparison within three separate blocks (green scale for IVC, red scale for ALB and blue scale for PYR). The second track depicts the difference values (∆PSI) of higher (red) and lower (blue) DASes in each compared group. The third track presents the types of alternative splicing events, where green represents SEs, blue represents A3SSs, yellow represents A5SSs and purple represents MXEs. The inner track shows the correlation links between each group, where pink links depict intersected genes for IVC and PYR DASes, gold depict intersected genes for ALB and PYR DAS events and blue links depict intersected genes after ALB and IVC treatment. A3SS, Alternative 3′ splice site' A5SS, alternative 5′ splice site; ALB, albendazole; AS, alternative splice; DASes, differential alternative splicing events; IVC, ivermectin; MXE, mutually exclusive exons; PSI, percent of splicing inclusion; PYR, pyrantel; SE, skipped intron; RI, retained intron

A comparable number of ASEs were detected across treatments, with 2997, 2992 and 2711 ASEs under the ALB, PYR and IVC treatments, respectively. However, the number of DASes varied, being highest in the IVC treatment group (*n* = 15), followed by the PYR treatment group (7) and the ALB treatment group (5) (Fig. 2; Additional file 2: Table S9). In the ALB-treated group, five significant DASes were identified, including A3SS in ASIM_0001014601 (*ZC3H12A*; ΔIncLevel = – 0.223; FDR = 0.024), and SE events in genes encoding a trehalase-like protein, a RING finger protein and a moesin/ezrin/radixin-like protein. An unannotated gene (MSTRG.17504) also exhibited A3SS (ΔIncLevel =  + 0.548; FDR = 0.01), indicating regulation of stress, protein degradation and cytoskeletal organization (Fig. 2; Additional file 1: Figure S5; Additional file 2: Table S9). The IVC group showed the widest ΔPSI range (+ 0.616 to – 0.556) and included splicing alterations in genes involved in RNA processing, membrane transport and intracellular signaling. Notable examples were SE events in ASIM_0001057901, ASIM_0001291601, and A5SS in MSTRG.17302. Recurring A3SS and A5SS events were found in MSTRG.13784 (*zinc finger protein*; ΔIncLevel = – 0.315) and MSTRG.21923 (*PH domain-containing protein*; ΔIncLevel =  + 0.609) (Fig. 2; Additional file 1: Figure 6; Additional file 2: Table S9). In the PYR group, seven DASes were observed, including SE in ASIM_0001079101 (ΔIncLevel = – 0.583; FDR = 0.01) and A5SS in MSTRG.16329 (ΔIncLevel =  + 0.265) (Fig. 2; Additional file 1: Figure S7; Additional file 2: Table S9). Splicing changes affected genes associated with ion transport (MSTRG.12187), extracellular matrix remodeling (MSTRG.20748) and metabolite transport (MSTRG.15994, MXE in YwtG-like transporter) (Fig. 2; Additional file 2: Table 9).

### Allele-specific variant profiling and transcriptomic responses to ALB, IVC and PYR

**Fig. 3:**
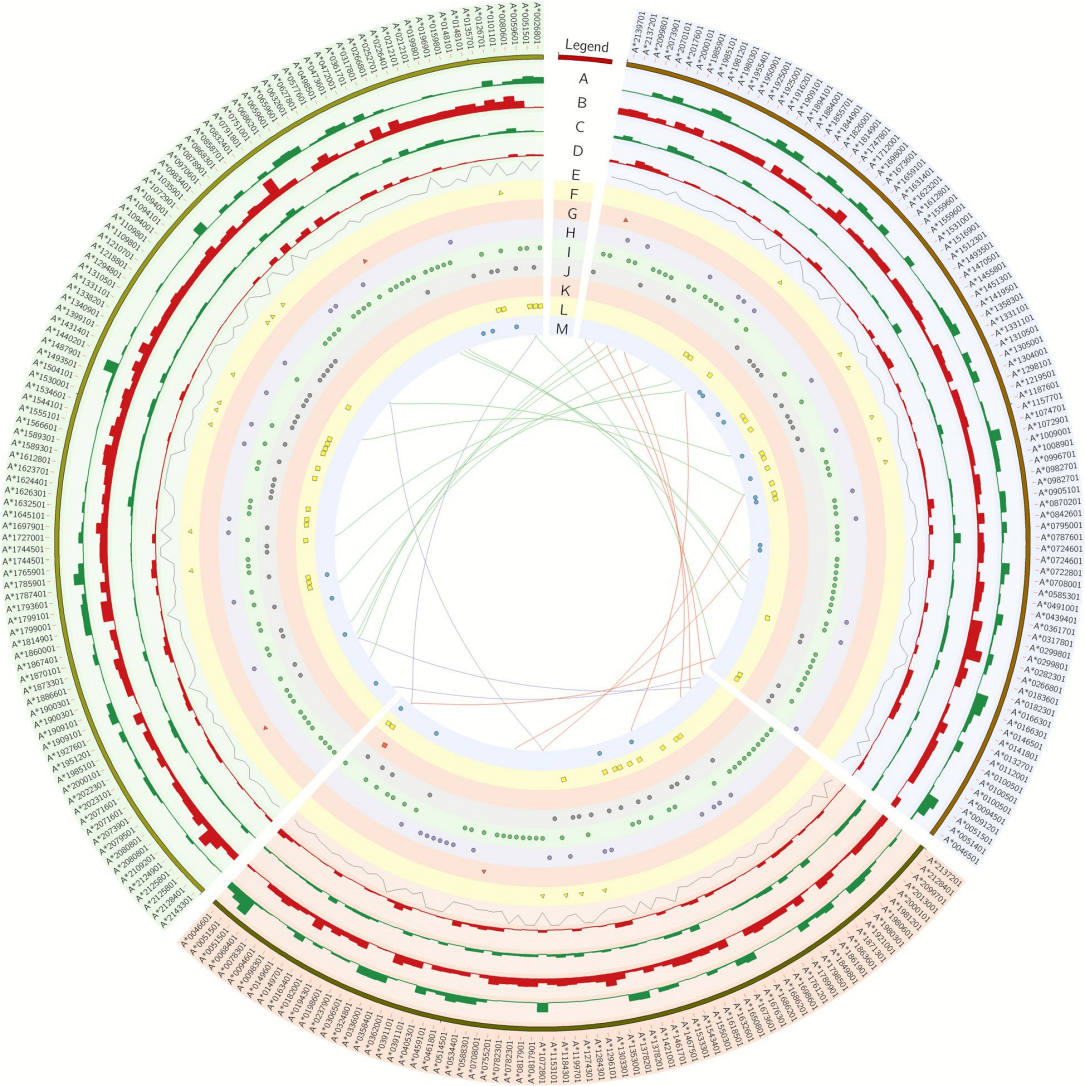
A circular chart illustrates the associations between single nucleotide variants (SNVs) across three comparisons: ivermectin (IVC; green background), albendazole (ALB; red background) and pyrantel (PYR; blue background). The outermost track depicts the *Anisakis simplex* gene accessions. The histogram tracks display the frequency of alternative and reference variants within treated samples (*A*,* C*) and control samples (*B*,* D*). Track* E* presents a line graph of the FST indicator (threshold > 0.66). The subsequent eight middle scatter tracks represent SNVs located within the upstream gene region (*F*), 5′ untranslated region (UTR) (*G*), those causing missense effects (*H*), those causing synonymous effects (*I*), intron regions (*J*), 3’ UTR regions (*K*), downstream gene regions (*L*) and other modification types (*M*). The innermost track shows the correlation links between experimental groups. Red links indicate intersected genes with SNVs for trials involving ALB and PYR, green links describe intersected genes with SNVs for trials involving IVC and PYR and blue links illustrate intersected genes with SNVs for trials involving ALB and IVC

In the experimental model, 603,522, 566,305 and 591,284 SNVs were identified following the in vitro exposure of larvae to ALB, IVC and PYR, respectively. After allele frequency comparisons between the experimental (treatment) samples and CTR, 3273, 3086 and 1794 outliers (heterozygous positions) for ALB, IVC and PYR were followed to the next stage of analysis. The downstream filtration steps (sum of coverage for each position > 30) qualified 74 SNVs after ALB treatment. After the application of IVC, the same filtration step as that for* Anisakis* transcriptome screening revealed 107 substitutions that changed its allele frequency. Analysis of the third experimental anthelmintic (PYR) identified 92 SNVs as potential candidates of allelic specific variants. Nine, four and one of the SNVs had similar allelic frequency changes between IVC/PYR, ALB/PYR and ALB/IVC, respectively. Three of the significant substitutions were common for all treatments applied. The 68 protein coding genes were potential candidate targets of transcriptomic response for the ALB treatment. The two other anthelmintics involved transcriptomic modulations of 83 (PYR treatment) and 95 (IVC treatment) protein coding transcripts. Based on genomic localization, the highest imbalance of allele expression between the ALB/PYR/IVC-treated groups and the* Anisakis* CTR were detected within A-to-G and C-to-T substitutions. SnpEff assigned allelic-specific expression candidates to the following main variant consequences: upstream gene variants (n = 8), 5′ UTR variants (n = 4), 3′ UTR variants (n = 1), missense (n=32), synonymous (n=131, intron variant calling (n=71), stop lost (n=2), splice region variant (n=13), start retained variant/stop retained variant (n=1) and downstream variant (n=20). S—s. (Fig. 3; Additional file 2: Table S10).

### Allele frequency validation using nanopore sequencing

We identified 29 SNVs among three drug-treated groups (IVC, ALB, PYR) and their respective CTR group. Each SNV is defined by its contig position, reference nucleotide (Ref), alternate nucleotide (Alt) and variant allele frequency (VAF), evaluated in both the treated and CTR groups. In all three treatment groups, SNVs demonstrated significant alterations in allele frequency between the treated groups and the CTR. In the IVC experiment, six out of 10 SNVs demonstrated reduced VAF in treated samples compared to the CTR (e.g. ASIM_scaffold0000920:14012 A → C), whereas four SNVs displayed elevated VAF following treatment (e.g. ASIM_contig0004408:1203 C → T). Similar patterns of enrichment and depletion of variant alleles were seen in the ALB and PYR trials (Additional file 2: Table S11). Both a reference and an alternative variant were identified for all SNV positions detected, confirming the presence of a heterozygous SNV (genotype 0/1). Nanopore sequencing allowed the estimation of the presence of variant alleles (VAF, frequency allele), with many positions showing an almost even distribution (approx.  50:50), confirming their heterozygous character and allowing further analysis of allele-specific gene expression. The identification of SNVs in regions affecting splicing (e.g. SPLICE region, STOP/SPLICE) enabled recognition of potential functional changes in transcript modification. These findings confirm the presence of polymorphic areas in genomic DNA linked to RNA polymorphisms, demonstrating drug-specific variations in allele frequencies indicative of selecting pressures (Additional file 2: Table S11).

### Multiomic analysis of drug impact on GO terms related to immune system response, cuticle development and neuron projection guidance in *A. simplex*

**Fig. 4:**
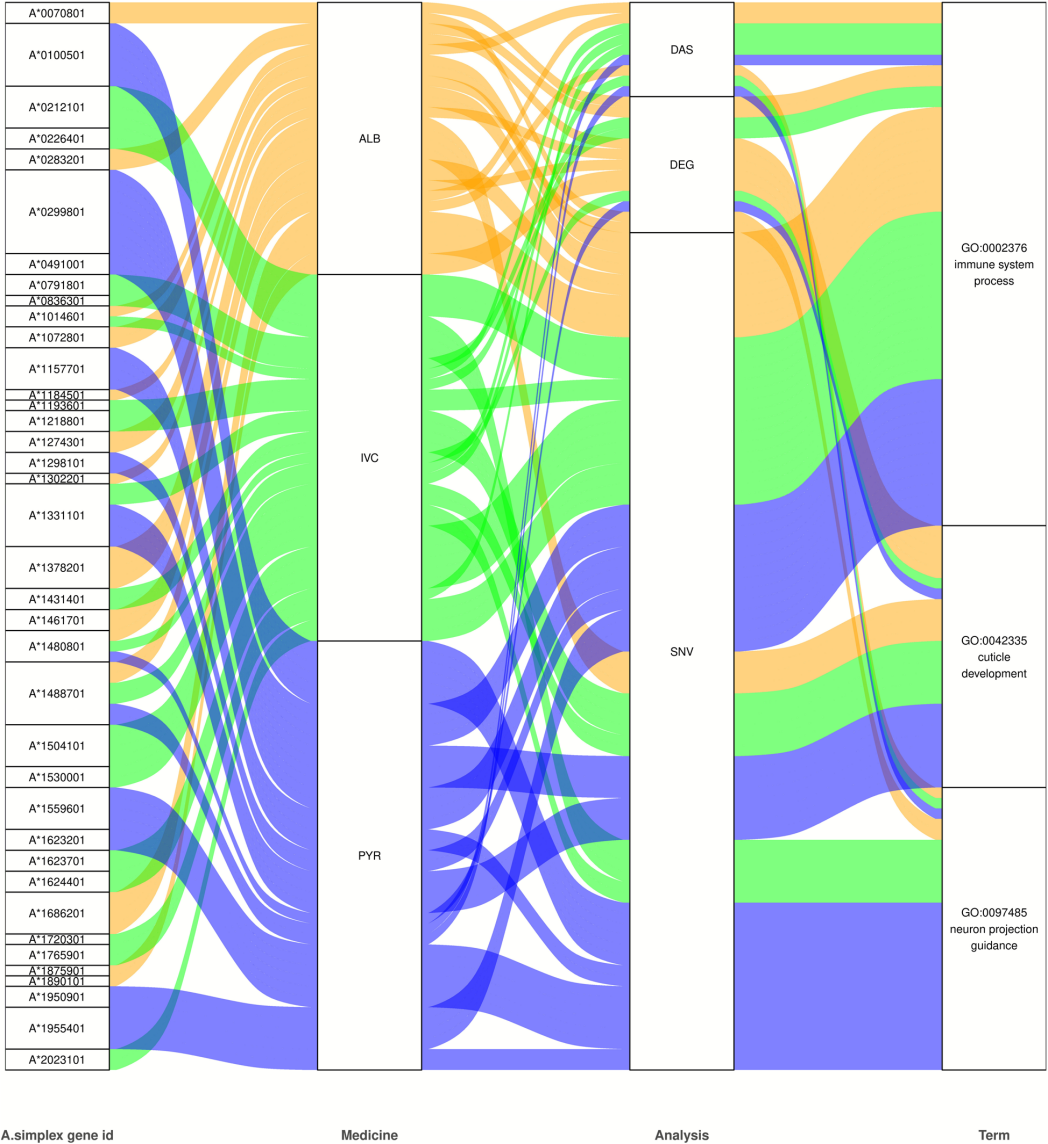
Sankey diagram illustrating the relationships between the identified GO terms, DEGs, DASes and SNVs. The “*A. simplex* gene id” column provides gene identifiers. The “Medicine” column corresponds to the drug in which a significant result was detected. The “Analysis” column contains information about the molecule, indicating whether it was DEG, DAS or SNV. The “Term” column corresponds to the selected four GO processes. Blue links are correspond to PYR, orange links correspond to ALB and green links correspond to IVC. ALB, Albendazole; DASes, differential alternative splicing events; DEGs, differentially expressed genes; GO, gene ontology; IVC, ivermectin; PYR, pyrantel; SNV, single nucleotide variant

The GO terms were assigned to the *A. simplex* transcripts. Three processes were focused on: immune system process (GO:0002376), cuticle development (GO:0042335) and neuron projection guidance (GO:0097485). Multiomics regulations of these three processes were identified by combining results from the SNV, DEG and DAS analyses (Fig. 4; Additional file 1: Table S5). In the case of cuticle development, ALB, IVC and PYR were found to differ in the number of molecules influencing this process. IVC and PYR drugs were associated with a larger number of molecules related to cuticle development, while ALB showed a slightly lower diversity of molecules. For example, ALB was linked to five molecules related to DEGs and one molecule related to SNVs, whereas IVC contained one molecule related to DEGs and 3 molecules related to SNVs. PYR was associated with one molecule related to DEGs and two molecules related to SNVs. Regarding immune system processes, the drugs ALB, IVC and PYR also differed in the number of molecules influencing this process. ALB was linked to two molecules associated with DASes and DEGs, as well as four molecules related to SNVs. For IVC, the number of molecules varied, including three molecules related to DASes, two molecules related to DEGs and seven molecules related to SNVs. PYR contained one molecule related to DASes and six molecules related to SNVs. In the case of neuron projection guidance, the drugs ALB, IVC and PYR also differed in the number of molecules influencing this process. ALB was linked to one molecule related to DASes and two molecules related to DEGs; IVC contained one molecule related to DASes and three molecules related to SNVs; and PYR was associated with one molecule related to DASes and five molecules related to SNVs. In summary, the drugs IVC and PYR contained significantly more molecules associated with various biological processes, particularly in the context of processes such as cuticle development, immune system and neuron projection guidance. ALB exhibited a lower diversity of molecules in these processes, which may indicate the specific mechanisms of action for each drug (Fig. 4; Additional file 2: Table S12).

## Discussion

Anthelmintic resistance in parasitic nematodes is a multifactorial phenomenon driven by genetic, transcriptomic and epigenetic mechanisms. Recent studies have identified several genetic factors contributing to resistance, including differential gene expression, SNPs and alternative splicing events. In the red stomach worm *Haemonchus contortus*, comparisons between resistant and susceptible strains revealed distinct gene expression patterns, although high genetic variability complicates interpretation of the results [50]. Moreover, lncRNAs have been linked to ALB resistance in this nematode species [22], while SNPs affecting splicing have been implicated in gastrointestinal nematode resistance in sheep, particularly in immune and sterol metabolism pathways [24]. In the brown stomach worm *Teladorsagia circumcincta*, upregulation of the P-glycoprotein gene *Tci-pgp-9* in all life stages, especially in eggs, suggests a role in IVC resistance [12]. In the present study, we applied high-throughput transcriptomic and variant analysis to investigate the response of *Anisakis simplex* s.s. L3 larvae to three anthelmintic compounds: ALB, IVC and PYR. We identified drug-specific transcriptomic signatures related to cuticle formation, neuronal development and immune processes, with ALB mainly regulating cuticle-associated genes through DEGs, IVC inducing AS in immune-related genes and PYR causing numerous SNVs across all functional categories. These results suggest both overlapping and distinct mechanisms of drug response in *A. simplex*. Differential gene expression in the treated larvae affected genes involved in cuticle biosynthesis, oxidative stress response and xenobiotic metabolism—findings consistent with those of studies in other nematodes [51–53]. Notably, we detected exon skipping in *trehalase* and complex splicing (SE/A5SS) in *moesin/ezrin/radixin-like protein 1 (MERP1)* in larvae exposed to ALB and IVC. Although not previously reported in nematodes, such ASes are reminiscent of resistance-related splicing changes in *H. contortus* (e.g. *acr-8*) [54–56] and apicomplexan parasites [57] and efflux pump regulation in fungi under antifungal stress [58]. In *A. simplex*, trehalase AS may impact energy homeostasis, while MERP1 may influence cytoskeletal remodeling during drug stress. Notably, the threonine residue (merlin T251, ezrin T235, radixin T235, moesin T235), which is conserved across all merlin–ezrin–radixin–moesin (ERM) proteins, is conservatively substituted by serine in nematode ERMs [59]. We also identified a splice-region SNV in trehalose phosphatase, a key enzyme in trehalose biosynthesis. In the roundworm *Caenorhabditis elegans*, deletion of its ortholog *gob-1* is embryonically lethal [60, 61] and in the nematode *Brugia malayi*, the trehalose pathway is upregulated in infective stages [62]. The co-occurrence of SNVs and ASes in this pathway suggests stress-induced destabilization of trehalose metabolism, potentially as a strategy to redirect energy use or counter osmotic and oxidative stress. These results align with our previous work demonstrating changes in trehalose synthesis enzyme activity in *A. simplex* following drug treatment [63, 64]. We cross-referenced our key findings (e.g., *trehalase, trehalose phosphatase, ABCA2, MERP1*) with the WormBase ParaSite and confirmed the conservation of orthologs and splice-associated domains [65]. Importantly, Variant Effect Predictor (VEP) annotations indicated that SNVs occurred in or near conserved splice motifs, further supporting their potential regulatory relevance [66]. Our SNV analysis revealed multiple missense mutations in genes involved in detoxification (e.g. *ACSL5, serine carboxypeptidase, PEPCK*), oxidative stress (e.g. *SOD1, cyclophilin*), drug transport (e.g. *ABCA2, MFS10*), gene regulation (e.g. RNA-binding proteins, *ZNF, WDR4*), cytoskeletal organization (*dynein, ZIP transporter*) and genome stability (*integrase-like, RIH-domain proteins*). These findings are consistent with previous reports linking ABC transporter mutations to IVC resistance in *H. contortus* and *Onchocerca volvulus *[67–71]. Similarly, oxidative stress genes like *SOD1* and *glutathione S-transferases* are involved in the response to IVC and PYR in *Pseudoterranova* and *Contracaecum *[8, 16]. Regulatory and non-coding variants add a layer of post-transcriptional control. In our dataset, we detected 71 intronic and 13 splice-region SNVs affecting genes involved in metabolism, signaling and stress response. Intronic variants may interfere with splicing enhancers or silencers, while SNVs within canonical splice sites can abrogate proper exon–intron recognition, potentially altering transcript stability or isoform diversity [66]. Among the genes impacted by these variants were *ABC transporters*, *oxidoreductases*, *serine carboxypeptidase S28* and *HDAC1*, all of which are implicated in drug efflux, detoxification, and epigenetic regulation. Such changes could have functional consequences for xenobiotic metabolism, contributing to altered drug susceptibility or resistance phenotypes [72–74]. The strong positive associations with specific mRNA genes identified in the correlation analysis of the lncRNA transcript suggest the existence of drug-specific regulatory mechanisms. In the response to ALB, the correlation of MSTG.1471 with transcript AT04676p (protein with a putative function in the cytoskeleton) and of MSTRG.1342 with *O-acyltransferase* may indicate the involvement of this lncRNA in cytoskeletal homeostasis and lipid metabolism under conditions of structural destabilization by ALB [75, 76]. In the case of IVC, correlations of the DELs MSTRG.4373 and MSTRG.886 with the enzyme of the methionine salvage pathway (*1,2-dihydroxy-3-keto-5-methylthiopentene dioxygenase*) as well as between MSTG.1095 and MSTRG.5177 and a BPI2 *domain-containing protein* suggest the activation of detoxification mechanisms and prevention of oxidative stress [7, 77, 78]. In response to PYR, the association of MSTG.886 with a metalloendopeptidase may reflect involvement in proteolytic and adaptive processes in target tissues of the drug, such as neurons and muscles [79]. Additionally, 5′/3′ UTR and up- or downstream SNVs were found in genes related to signaling (*Piezo1*), translation (ribosomal S7, *RFC*), vesicle transport (*kinesin*,* Tomosyn-like*) and proteostasis (DnaJ, interferon-like proteins), suggesting roles in transcript stability, translation efficiency or transcriptional regulation [80]. These findings support the theory that lncRNAs and non-coding regulatory regions are involved in modulating the transcriptomic plasticity of *A. simplex* in response to drug exposure, possibly through mechanisms analogous to those in other helminths [81, 82].

Although our findings provide valuable insights, they are based on cross-sectional data and require validation in larger, longitudinal cohorts to capture gene expression dynamics over time. Future studies should include more larvae to strengthen generalizability. SNV calling was performed on RNA-Seq reads mapped to the *A. simplex* reference genome rather than on DNA-Seq data aligned to a complete genomic assembly. As a result, these variant calls may be affected by mapping artifacts, RNA editing, allele-specific expression and incomplete transcript representation and should therefore be interpreted with caution. Nonetheless, a key strength of our study is that, even with only four larvae per group, we detected consistent and biologically meaningful differences between groups. Integrating metabolomic and lipidomic data could further provide a comprehensive systems-level understanding of *A. simplex* responses to drug treatment.

## Conclusions

While our study provides the first integrative transcriptomic analysis of *Anisakis simplex* L3 larvae exposed to three anthelmintics (ALB, IVC, and PYR), several limitations shape the interpretation of our results and define future research priorities. A key constraint in *Anisakis* research is the inability to culture resistant and susceptible isolates, making classical resistance models inapplicable. Consequently, rather than comparing resistant versus susceptible strains, future studies should focus on functional validation of the candidate molecular markers identified in the present study (e.g. SNVs in trehalose metabolism, ABC transporters or splicing-related genes). Approaches such as RNAi knockdown or heterologous expression in model nematodes (e.g. *C. elegans*) could help test the impact of these variants on drug sensitivity. Moreover, while long-read nanopore sequencing enabled detection of full-length isoforms and haplotypes, its error rates in homopolymer regions may limit SNV accuracy. Refining these data with hybrid sequencing or improved basecalling algorithms could enhance variant resolution. To further understand adaptive responses, future research should: (i) apply time-course transcriptomic profiling to distinguish between early stress responses and stable transcriptomic remodeling; (ii) explore epigenetic and ncRNA mechanisms, given the observed lncRNA–mRNA correlations with drug-response genes; (iii) incorporate proteomic and metabolomic data to link gene regulation with parasite physiology and fitness under drug pressure; and (iv) investigate environmentally driven population variability to infer natural selection signatures associated with drug exposure in the wild. In the absence of experimental selection, these integrative, multi-omics and functional approaches offer the most promising path forward to elucidate the molecular basis of drug tolerance and support surveillance strategies in zoonotic marine nematodes. The identification of drug-specific transcriptomic signatures and regulatory elements in *A. simplex* provides a foundation for the rational design of next-generation anthelmintics. Candidate pathways such as trehalose metabolism, ABC transporter systems, alternative splicing machinery and cytoskeletal remodeling proteins emerged as key nodes of drug response and may serve as novel therapeutic targets. Furthermore, SNVs and expression profiles linked to detoxification or stress responses can inform the development of drug combinations that circumvent compensatory resistance mechanisms. The lncRNA–mRNA correlations also highlight the potential of targeting ncRNA-mediated regulation as an innovative antiparasitic strategy. These insights are particularly valuable for zoonotic marine nematodes, where drug development is challenged by a lack of genomic resources and experimental models, and could guide the discovery of compounds with enhanced efficacy or broader antihelminthics activity.

## Supplementary Information

Additional file 1: Figure S1. The Venn diagram illustrates the number of DEGs and the common DEGs in the ALB, IVC, PYRcolor comparisons.** Figure S2. **The Venn diagram illustrates the number of DELs and the common DELs in the ALB, IVC, PYRcolor comparisons.** Figure S3. **The results of correlations analysis between RT-PCR method and RNA-Seq. Selected DEGs were marked with different color for each treatment comparison. The* Y*-axis shows the log2 obtained by the RNA-seq method, while the* X*-axis shows the log2 of validated DEGs measured by the qPCR method. Details can be found in Additional file 2: Table S2.** Figure S4.** Relative expression levels of genes in response *A*.* simplex*to drug treatmentscompared to control, as determined by quantitative real-time PCR. mRNA expression of each genewas normalized to housekeeping gene expression and expressed relative to the control group. Data are presented as mean ± SEM from* n* = 4 samples. Statistical significance is indicated as* p*-values interpreted as follows: 0.0332, 0.0021, 0.0002, and <0.0001.^2045201,^1890101,^4133,^1857601,^1723901,^1578201,^1493901,^1486201,^1480801,^1418601,^1260001,^836301,^307801,^221901, ^74501,^22701.** Figure S5. **Volcano plot depicts the PSI levels forin the ALB experimental comparison. The* X*-axis represents the difference in PSI valuesfor each ASE, while the* Y*-axis displays the negative logarithmic FDR. A horizontal dotted line indicates the negative logarithmic value of the FDR cutoff, and two vertical lines represent the absolute ΔPSI value of 0.1. Colored points denote different types of significant DASes, and gray points represent non-significant DAS events**. Figure S6. **Volcano plot illustrating the PSI levels for significant DASes in the IVC experimental comparison. The* X*-axis displays the difference in PSI valuesfor each DASes, and the* Y*-axis shows the negative logarithmic FDR. A horizontal dotted line marks the negative logarithmic value of the FDR cutoff, while two vertical lines indicate the absolute ΔPSI value of 0.1. Colored points represent distinct types of significant DAS events, and gray points represent non-significant ASEs.** Figure S7. **Volcano plot illustrates the percentage of inclusionlevels for significant DAS events in the PYR experimental comparison. The* X*-axis displays the difference in PSI valuesfor each ASE, and the* Y*-axis shows the negative logarithmic FDR. A horizontal dotted line marks the negative logarithmic value of the FDR cutoff, while two vertical lines indicate the absolute ΔPSI value of 0.1. Colored points represent distinct types of significant DAS events, and gray points represent non-significant DAS events.

Additional file 2: Table S1. Detailed bioinformatics workflow. **Table S2.** Comparison of log2FoldChange values between RNA-seq and real timePCR.. **Table S3.** The table summarizes RNA-seq quality and yield across 16 samples.. **Table S4.** Differentially expressed genes identified in A. simplex after three medicine treatment . **Table S5.** Differentially expressed long nonocoding RNA identified in A. simplex after three medicine treatment . **Table S6.** Correlation between DELs and DEGs after Albendazol treatment. **Table S7.** Correlation between DELs and DEGs after Ivermectin treatment. **Table S8.** Correlation between lncRNA and DEGs after Pyrantel treatment. **Table S9. DAS events identified after Albendazol, Ivermectin and Pyrantel treatment**. **Table S10. SNVs indicated difference in allele specific expression (ASE)**. **Table S11.Single nucleotide variation on DNA seqeunces by Nanopore seqeuncing** . **Table S12.** DEGs, DASs and SNVs assignment to GO terms..

## Data Availability

Transcriptomic data are available at the European Nucleotide Archive (ENA) under accession number PRJEB98681.

## References

[1] Matoušková P, Vokřál I, Lamka J, Skálová L. The role of xenobiotic-metabolizing enzymes in anthelmintic deactivation and resistance in helminths. Trends Parasitol. 2016;32:481–91. 10.1016/j.pt.2016.02.004. 26968642 10.1016/j.pt.2016.02.004

[2] Buchmann K, Mehrdana F. Effects of anisakid nematodes *Anisakis simplex* (s.l.), *Pseudoterranova decipiens* (s.l.) and *Contracaecum osculatum* (s.l.) on fish and consumer health. Food Waterborne Parasitol. 2016;4:13–22. 10.1016/j.fawpar.2016.07.003.

[3] Sohn W-M, Na B-K, Kim TH, Park T-J. Anisakiasis: report of 15 gastric cases caused by* Anisakis *type I larvae and a brief review of Korean anisakiasis cases. Korean J Parasitol. 2015;53:465–70. 10.3347/kjp.2015.53.4.465. 26323845 10.3347/kjp.2015.53.4.465 PMC4566497

[4] Audicana MT.* Anisakis*, something is moving inside the fish. Pathogens. 2022. 10.3390/pathogens11030326. 35335650 10.3390/pathogens11030326 PMC8950136

[5] Baird FJ, Gasser RB, Jabbar A, Lopata AL. Foodborne anisakiasis and allergy. Mol Cell Probes. 2014;28:167–74. 10.1016/j.mcp.2014.02.003. 24583228 10.1016/j.mcp.2014.02.003

[6] Whittaker JH, Carlson SA, Jones DE, Brewer MT. Molecular mechanisms for anthelmintic resistance in strongyle nematode parasites of veterinary importance. J Vet Pharmacol Ther. 2017;40:105–15. 10.1111/jvp.12330. 27302747 10.1111/jvp.12330

[7] James CE, Hudson AL, Davey MW. Drug resistance mechanisms in helminths: is it survival of the fittest? Trends Parasitol. 2009;25:328–35. 10.1016/j.pt.2009.04.004. 19541539 10.1016/j.pt.2009.04.004

[8] Stryiński R, Polak I, Gawryluk A, Rosa P, Łopieńska-Biernat E. The response of *Anisakis simplex* (s. s.) to anthelmintics—specific changes in xenobiotic metabolic processes. Exp Parasitol. 2024;261:108751. 10.1016/j.exppara.2024.108751. 38604302 10.1016/j.exppara.2024.108751

[9] Mladineo I, Trumbić Ž, Hrabar J, Vrbatović A, Bušelić I, Ujević I, et al. Efficiency of target larvicides is conditioned by ABC-mediated transport in the zoonotic nematode* Anisakis pegreffii.* Antimicrob Agents Chemother. 2018;62:1–14. 10.1128/AAC.00916-18. 10.1128/AAC.00916-18 PMC6125575 29987147

[10] Łopieńska-Biernat E, Stryiński R, Paukszto Ł, Jastrzębski JP. Correlation of NHR-48 transcriptional modulator expression with selected CYP genes’ expression during thiabendazole treatment of *Anisakis simplex* (s.l.)?—an in vitro study. Pathogens. 2020;9:1030. 10.3390/pathogens9121030. 33316888 10.3390/pathogens9121030 PMC7764245

[11] Jones LM, Flemming AJ, Urwin PE. NHR-176 regulates cyp-35d1 to control hydroxylation-dependent metabolism of thiabendazole in* Caenorhabditis elegans*. Biochem J. 2015;466:37–44. 10.1042/BJ20141296. 25406993 10.1042/BJ20141296

[12] Dicker AJ, Nisbet AJ, Skuce PJ. Gene expression changes in a P-glycoprotein (Tci-pgp-9) putatively associated with ivermectin resistance in *Teladorsagia circumcincta*. Int J Parasitol. 2011;41:935–42. 10.1016/j.ijpara.2011.03.015. 21683705 10.1016/j.ijpara.2011.03.015

[13] Fissiha W, Kinde MZ. Anthelmintic resistance and its mechanism: a review. Infect Drug Resist. 2021;14:5403–10. 10.2147/IDR.S332378. 34938088 10.2147/IDR.S332378 PMC8687516

[14] Bao M, Pierce GJ, Strachan NJC, Pascual S, González-Muñoz M, Levsen A. Human health, legislative and socioeconomic issues caused by the fish-borne zoonotic parasite Anisakis: challenges in risk assessment. Trends Food Sci Technol. 2019;86:298–310. 10.1016/j.tifs.2019.02.013.

[15] Jurado-Palomo J, López-Serrano MC, Moneo I. Multiple acute parasitization by *Anisakis simplex*. J Investig Allergol Clin Immunol. 2010;20:437–41. 20945613

[16] Polak I, Stryiński R, Podolska M, Pawlak J, Bittner MW, Wiśniewski G, et al. Drug efficacy on zoonotic nematodes of the Anisakidae family: new metabolic data. Parasitology. 2022;149:1065–77. 10.1017/S0031182022000543. 35443901 10.1017/S0031182022000543 PMC10090616

[17] Pacios E, Arias-Diaz J, Zuloaga J, Gonzalez-Armengol J, Villarroel P, Balibrea JL. Albendazole for the treatment of anisakiasis ileus. Clin Infect Dis. 2005;41:1825–6. 10.1086/498309. 16288416 10.1086/498309

[18] Gómez-Mateos M, Arrebola F, Navarro MC, Romero MC, González JM, Valero A. Acute anisakiasis: pharmacological evaluation of various drugs in an animal model. Dig Dis Sci. 2021;66:105–13. 10.1007/s10620-020-06144-2. 32107679 10.1007/s10620-020-06144-2

[19] Polak I, Łopieńska-Biernat E, Stryiński R, Mateos J, Carrera M. Comparative proteomics analysis of *Anisakis simplex* s.s.—evaluation of the response of invasive larvae to ivermectin. Genes. 2020;11:710. 10.3390/genes11060710. 32604878 10.3390/genes11060710 PMC7349835

[20] Tuersong W, Zhou C, Wu S, Qin P, Wang C, Di W, et al. Comparative analysis on transcriptomics of ivermectin resistant and susceptible strains of Haemonchus contortus. Parasit Vectors. 2022;15:159. 10.1186/s13071-022-05274-y. 35524281 10.1186/s13071-022-05274-y PMC9077910

[21] Abubucker S, McNulty SN, Rosa BA, Mitreva M. Identification and characterization of alternative splicing in parasitic nematode transcriptomes. Parasit Vectors. 2014;7:151. 10.1186/1756-3305-7-151. 24690220 10.1186/1756-3305-7-151 PMC3997825

[22] Chen X, Wang T, Guo W, Yan X, Kou H, Yu Y, et al. Transcriptome reveals the roles and potential mechanisms of lncRNAs in the regulation of albendazole resistance in* Haemonchus contortus.* BMC Genomics. 2024;25:188. 10.1186/s12864-024-10096-6. 38368335 10.1186/s12864-024-10096-6 PMC10873934

[23] Hagiwara M. Alternative splicing: a new drug target of the post-genome era. Biochem Biophys Acta. 2005;1754:324–31. 10.1016/j.bbapap.2005.09.010. 16260193 10.1016/j.bbapap.2005.09.010

[24] Cunha SMF, Willoughby OB, Schenkel FS, Mallard B, Karrow NA, Cánovas A. 219 Identification of functional single nucleotide polymorphisms responsible for alternative splicing affecting gastrointestinal nematodes resistance in grazing sheep. J Anim Sci. 2023;101:138–9. 10.1093/jas/skad281.168.

[25] Iglesias L, Valero A, Benítez R, Adroher FJ. In vitro cultivation of *Anisakis simplex*: pepsin increases survival and moulting from fourth larval to adult stage. Parasitology. 2001;123:285–91. 10.1017/S0031182001008423. 10.1017/s0031182001008423 11578092

[26] Martin F, Dube F, Lindsjö OK, Eydal M, Höglund J, Bergström T, et al. Transcriptional responses in* Parascaris univalens* after in vitro exposure to ivermectin, pyrantel citrate and thiabendazole. Parasit Vectors. 2020. 10.21203/rs.3.rs-17857/v1. 10.1186/s13071-020-04212-0 PMC7346371 32646465

[27] Hu Y, Ellis BL, Yiu YY, Miller MM, Urban JF, Shi LZ, et al. An extensive comparison of the effect of anthelmintic classes on diverse nematodes. PLoS ONE. 2013;8:e70702. 10.1371/journal.pone.0070702. 23869246 10.1371/journal.pone.0070702 PMC3712009

[28] Fasanmade AA, Akanni AO, Olaniyi AA, Fasanmade AA, Tayo F. Bioequivalence of pyrantel pamoate dosage forms in healthy human subjects. Biopharm Drug Dispos. 1994;15:527–34. 10.1002/bdd.2510150610. 7993990 10.1002/bdd.2510150610

[29] Alvarez L, Mottier M, Sánchez S, Lanusse C. Ex vivo diffusion of albendazole and its sulfoxide metabolite into* Ascaris suum* and* Fasciola hepatica*. Parasitol Res. 2001;87:929–34. 10.1007/s004360100471. 11728018 10.1007/s004360100471

[30] Dayan AD. Albendazole, mebendazole and praziquantel. Review of non-clinical toxicity and pharmacokinetics. Acta Trop. 2003;86:141–59. 10.1016/S0001-706X(03)00031-7. 12745134 10.1016/s0001-706x(03)00031-7

[31] González Canga A, Sahagún Prieto AM, Diez Liébana MJ, Fernández Martínez N, Sierra Vega M, García Vieitez JJ. The pharmacokinetics and interactions of ivermectin in humans—a mini-review. AAPS J. 2008;10:42–6. 10.1208/s12248-007-9000-9. 18446504 10.1208/s12248-007-9000-9 PMC2751445

[32] Bolger AM, Lohse M, Usadel B. Trimmomatic: a flexible trimmer for Illumina sequence data. Bioinformatics. 2014;30:2114–20. 10.1093/bioinformatics/btu170. 24695404 10.1093/bioinformatics/btu170 PMC4103590

[33] Dobin A, Davis CA, Schlesinger F, Drenkow J, Zaleski C, Jha S, et al. STAR: ultrafast universal RNA-seq aligner. Bioinformatics. 2013;29:15–21. 10.1093/bioinformatics/bts635. 23104886 10.1093/bioinformatics/bts635 PMC3530905

[34] Pertea M, Pertea GM, Antonescu CM, Chang T-C, Mendell JT, Salzberg SL. Stringtie enables improved reconstruction of a transcriptome from RNA-seq reads. Nat Biotechnol. 2015;33:290–5. 10.1038/nbt.3122. 25690850 10.1038/nbt.3122 PMC4643835

[35] Frazee AC, Pertea G, Jaffe AE, Langmead B, Salzberg SL, Leek JT. Ballgown bridges the gap between transcriptome assembly and expression analysis. Nat Biotechnol. 2015;33:243–6. 10.1038/nbt.3172. 25748911 10.1038/nbt.3172 PMC4792117

[36] Durinck S, Spellman PT, Birney E, Huber W. Mapping identifiers for the integration of genomic datasets with the R/Bioconductor package biomaRt. Nat Protoc. 2009;4:1184–91. 10.1038/nprot.2009.97. 19617889 10.1038/nprot.2009.97 PMC3159387

[37] Martin FJ, Amode MR, Aneja A, Austine-Orimoloye O, Azov AG, Barnes I, et al. Ensembl 2023. Nucleic Acids Res. 2023;51:D933–41. 10.1093/nar/gkac958. 36318249 10.1093/nar/gkac958 PMC9825606

[38] Pfaffl MW. A new mathematical model for relative quantification in real-time RT-PCR. Nucleic Acids Res. 2001;29:45e–45. 10.1093/nar/29.9.e45. 10.1093/nar/29.9.e45 PMC55695 11328886

[39] Shen S, Park JW, Lu Z, Lin L, Henry MD, Wu YN, et al. rMATS: Robust and flexible detection of differential alternative splicing from replicate RNA-Seq data. Proc Natl Acad Sci USA. 2014. 10.1073/pnas.1419161111. 25480548 10.1073/pnas.1419161111 PMC4280593

[40] Morgan M, Anders S, Lawrence M, Aboyoun P, Pagès H, Gentleman R. Shortread: a bioconductor package for input, quality assessment and exploration of high-throughput sequence data. Bioinformatics. 2009;25:2607–8. 10.1093/bioinformatics/btp450. 19654119 10.1093/bioinformatics/btp450 PMC2752612

[41] Li H. A statistical framework for SNP calling, mutation discovery, association mapping and population genetical parameter estimation from sequencing data. Bioinformatics. 2011;27:2987–93. 10.1093/bioinformatics/btr509. 21903627 10.1093/bioinformatics/btr509 PMC3198575

[42] Quinlan AR, Hall IM. BEDtools: a flexible suite of utilities for comparing genomic features. Bioinformatics. 2010;26:841–2. 10.1093/bioinformatics/btq033. 20110278 10.1093/bioinformatics/btq033 PMC2832824

[43] Weir BS, Cockerham CC. Estimating *F* -statistics for the analysis of population structure. Evolution. 1984;38:1358–70. 10.1111/j.1558-5646.1984.tb05657.x. 28563791 10.1111/j.1558-5646.1984.tb05657.x

[44] Cingolani P, Platts A, Wang LL, Coon M, Nguyen T, Wang L, et al. A program for annotating and predicting the effects of single nucleotide polymorphisms, SnpEff. Fly. 2012;6:80–92. 10.4161/fly.19695. 22728672 10.4161/fly.19695 PMC3679285

[45] Huerta-Cepas J, Szklarczyk D, Heller D, Hernández-Plaza A, Forslund SK, Cook H, et al. eggNOG 5.0: a hierarchical, functionally and phylogenetically annotated orthology resource based on 5090 organisms and 2502 viruses. Nucleic Acids Res. 2019. 10.1093/nar/gky1085. 30418610 10.1093/nar/gky1085 PMC6324079

[46] Gotz S, Garcia-Gomez JM, Terol J, Williams TD, Nagaraj SH, Nueda MJ, et al. High-throughput functional annotation and data mining with the Blast2GO suite. Nucleic Acids Res. 2008;36:3420–35. 10.1093/nar/gkn176. 18445632 10.1093/nar/gkn176 PMC2425479

[47] Wickham H. Data analysis. ggplot2: elegant graphics for data analysis. Springer International Publishing, Cham; 2016.

[48] Gu Z, Gu L, Eils R, Schlesner M, Brors B. Circlize implements and enhances circular visualization in R. Bioinformatics. 2014;30:2811–2. 10.1093/bioinformatics/btu393. 24930139 10.1093/bioinformatics/btu393

[49] Krzywinski M, Schein J, Birol İ, Connors J, Gascoyne R, Horsman D, et al. Circos: an information aesthetic for comparative genomics. Genome Res. 2009;19:1639–45. 10.1101/gr.092759.109. 19541911 10.1101/gr.092759.109 PMC2752132

[50] Rezansoff AM, Laing R, Martinelli A, Stasiuk S, Redman E, Bartley D, et al. The confounding effects of high genetic diversity on the determination and interpretation of differential gene expression analysis in the parasitic nematode *Haemonchus contortus*. Int J Parasitol. 2019;49:847–58. 10.1016/j.ijpara.2019.05.012. 31525371 10.1016/j.ijpara.2019.05.012

[51] Hartman JH, Widmayer SJ, Bergemann CM, King DE, Morton KS, Romersi RF, et al. Xenobiotic metabolism and transport in *Caenorhabditis elegans*. J Toxicol Environ Health B Crit Rev. 2021;24:51–94. 10.1080/10937404.2021.1884921. 33616007 10.1080/10937404.2021.1884921 PMC7958427

[52] Sandhu A, Badal D, Sheokand R, Tyagi S, Singh V. Specific collagens maintain the cuticle permeability barrier in *Caenorhabditis elegans*. Genetics. 2021. 10.1093/genetics/iyaa047. 33789349 10.1093/genetics/iyaa047 PMC8045729

[53] Goel V, Sharma S, Chakroborty NK, Singla LD, Choudhury D. Targeting the nervous system of the parasitic worm, *Haemonchus contortus* with quercetin. Heliyon. 2023;9:e13699. 10.1016/j.heliyon.2023.e13699. 36852031 10.1016/j.heliyon.2023.e13699 PMC9957779

[54] Fauvin A, Charvet C, Issouf M, Cortet J, Cabaret J, Neveu C. cDNA-AFLP analysis in levamisole-resistant* Haemonchus contortus* reveals alternative splicing in a nicotinic acetylcholine receptor subunit. Mol Biochem Parasitol. 2010;170:105–7. 10.1016/j.molbiopara.2009.11.007. 19932716 10.1016/j.molbiopara.2009.11.007

[55] Antonopoulos A, Doyle SR, Bartley DJ, Morrison AA, Kaplan R, Howell S, et al. Allele specific PCR for a major marker of levamisole resistance in* Haemonchus contortus*. Int J Parasitol. 2022. 10.1101/2022.04.08.487639. 10.1016/j.ijpddr.2022.08.001 PMC9399269 35970104

[56] Antonopoulos A, Charvet CL, Maitland K, Doyle SR, Neveu C, Laing R. Functional validation of novel levamisole resistance marker S168T in *Haemonchus contortus*. Int J Parasitol Drugs Drug Resist. 2024;24:100524. 10.1016/j.ijpddr.2024.100524. 38346379 10.1016/j.ijpddr.2024.100524 PMC10867575

[57] Yeoh LM, Lee VV, McFadden GI, Ralph SA. Alternative splicing in apicomplexan parasites. MBio. 2019. 10.1128/mBio.02866-18. 30782661 10.1128/mBio.02866-18 PMC6381282

[58] Lopes MER, Bitencourt TA, Sanches PR, Martins MP, Oliveira VM, Rossi A, et al. Alternative splicing in *Trichophyton rubrum* occurs in efflux pump transcripts in response to antifungal drugs. J Fungi. 2022;8:878. 10.3390/jof8080878. 10.3390/jof8080878 PMC9410333 36012866

[59] Michie KA, Bermeister A, Robertson NO, Goodchild SC, Curmi PMG. Two sides of the coin: Ezrin/Radixin/Moesin and Merlin control membrane structure and contact inhibition. Int J Mol Sci. 2019;20:1996. 10.3390/ijms20081996. 31018575 10.3390/ijms20081996 PMC6515277

[60] Kormish JD, McGhee JD. The *C. elegans* lethal gut-obstructed gob-1 gene is trehalose-6-phosphate phosphatase. Dev Biol. 2005;287:35–47. 10.1016/j.ydbio.2005.08.027. 16197937 10.1016/j.ydbio.2005.08.027

[61] Pellerone FI, Archer SK, Behm CA, Grant WN, Lacey MJ, Somerville AC. Trehalose metabolism genes in *Caenorhabditis elegans* and filarial nematodes. Int J Parasitol. 2003;33:1195–206. 10.1016/S0020-7519(03)00173-5. 13678635 10.1016/s0020-7519(03)00173-5

[62] Cross M, Rajan S, Chekaiban J, Saunders J, Hamilton C, Kim J-S, et al. Enzyme characteristics of pathogen-specific trehalose-6-phosphate phosphatases. Sci Rep. 2017;7:2015. 10.1038/s41598-017-02220-2. 28515463 10.1038/s41598-017-02220-2 PMC5435700

[63] Łopieńska-Biernat E, Paukszto, Jastrzębski JP, Makowczenko K, Stryiński R. Genes expression and in silico studies of functions of trehalases, a highly dispersed* Anisakis simplex* s. l. specific gene family. Int J Biol Macromol. 2019. 10.1016/j.ijbiomac.2019.02.042. 30779983 10.1016/j.ijbiomac.2019.02.042

[64] Łopieńska-Biernat E, Molcan T, Paukszto Ł, Jastrzębski JP, Myszczyński K. Modelling studies determing the mode of action of anthelmintics inhibiting in vitro trehalose-6-phosphate phosphatase (TPP) of *Anisakis simplex* s.l. Exp Parasitol. 2018;184:46–56. 10.1016/j.exppara.2017.11.006. 29170085 10.1016/j.exppara.2017.11.006

[65] Howe KL, Bolt BJ, Shafie M, Kersey P, Berriman M. WormBase ParaSite—a comprehensive resource for helminth genomics. Mol Biochem Parasitol. 2017;215:2–10. 10.1016/j.molbiopara.2016.11.005. 27899279 10.1016/j.molbiopara.2016.11.005 PMC5486357

[66] Kerins JA, Hanazawa M, Dorsett M, Schedl T. PRP-17 and the pre-mRNA splicing pathway are preferentially required for the proliferation versus meiotic development decision and germline sex determination in *Caenorhabditis elegans*. Dev Dyn. 2010;239:1555–72. 10.1002/dvdy.22274. 20419786 10.1002/dvdy.22274 PMC3097115

[67] Martin F, Eydal M, Höglund J, Tydén E. Constitutive and differential expression of transport protein genes in *Parascaris univalens* larvae and adult tissues after in vitro exposure to anthelmintic drugs. Vet Parasitol. 2021;298:109535. 10.1016/j.vetpar.2021.109535. 34340009 10.1016/j.vetpar.2021.109535

[68] Doyle SR, Bourguinat C, Nana-Djeunga HC, Kengne-Ouafo JA, Pion SDS, Bopda J, et al. Genome-wide analysis of ivermectin response by *Onchocerca volvulus* reveals that genetic drift and soft selective sweeps contribute to loss of drug sensitivity. PLoS Negl Trop Dis. 2017;11:e0005816. 10.1371/journal.pntd.0005816. 28746337 10.1371/journal.pntd.0005816 PMC5546710

[69] Mani T, Bourguinat C, Prichard RK. Polymorphism in ABC transporter genes of *Dirofilaria immitis*. Int J Parasitol Drugs Drug Resist. 2017;7:227–35. 10.1016/j.ijpddr.2017.04.004. 28494332 10.1016/j.ijpddr.2017.04.004 PMC5421822

[70] Mate L, Ballent M, Cantón C, Lanusse C, Ceballos L, Alvarez LLI, et al. ABC-transporter gene expression in ivermectin-susceptible and resistant* Haemonchus contortus* isolates. Vet Parasitol. 2022;302:109647. 10.1016/j.vetpar.2022.109647. 35065372 10.1016/j.vetpar.2022.109647

[71] Ardelli BF, Prichard RK. Identification of variant ABC-transporter genes among *Onchocerca volvulus* collected from ivermectin-treated and untreated patients in Ghana. West Afr Ann Trop Med Parasitol. 2004;98:371–84. 10.1179/000349804225003415. 10.1179/000349804225003415 15228718

[72] Jex AR, Liu S, Li B, Young ND, Hall RS, Li Y, et al. *Ascaris suum* draft genome. Nature. 2011;479:529–33. 10.1038/nature10553. 22031327 10.1038/nature10553

[73] Laing R, Kikuchi T, Martinelli A, Tsai IJ, Beech RN, Redman E, et al. The genome and transcriptome of *Haemonchus contortus*, a key model parasite for drug and vaccine discovery. Genome Biol. 2013;14:R88. 10.1186/gb-2013-14-8-r88. 23985316 10.1186/gb-2013-14-8-r88 PMC4054779

[74] Gilleard JS. Understanding anthelmintic resistance: the need for genomics and genetics. Int J Parasitol. 2006;36:1227–39. 10.1016/j.ijpara.2006.06.010. 16889782 10.1016/j.ijpara.2006.06.010

[75] Kotze AC, Hunt PW, Skuce P, von Samson-Himmelstjerna G, Martin RJ, Sager H, et al. Recent advances in candidate-gene and whole-genome approaches to the discovery of anthelmintic resistance markers and the description of drug/receptor interactions. Int J Parasitol Drugs Drug Resist. 2014;4:164–84. 10.1016/j.ijpddr.2014.07.007. 25516826 10.1016/j.ijpddr.2014.07.007 PMC4266812

[76] Laing R, Gillan V, Devaney E. Ivermectin—old drug, new tricks? Trends Parasitol. 2017;33:463–72. 10.1016/j.pt.2017.02.004. 28285851 10.1016/j.pt.2017.02.004 PMC5446326

[77] Selkirk ME, Smith VP, Thomas GR, Gounaris K. Resistance of filarial nematode parasites to oxidative stress. Int J Parasitol. 1998;28:1315–32. 10.1016/S0020-7519(98)00107-6. 9770616 10.1016/s0020-7519(98)00107-6

[78] Wang P, Xu J, Wang Y, Cao X. An interferon-independent lncRNA promotes viral replication by modulating cellular metabolism. Science. 1979;2017:1051–5. 10.1126/science.aao0409. 10.1126/science.aao0409 29074580

[79] Williamson SM, Storey B, Howell S, Harper KM, Kaplan RM, Wolstenholme AJ. Candidate anthelmintic resistance-associated gene expression and sequence polymorphisms in a triple-resistant field isolate of *Haemonchus contortus*. Mol Biochem Parasitol. 2011;180:99–105. 10.1016/j.molbiopara.2011.09.003. 21945142 10.1016/j.molbiopara.2011.09.003

[80] Barrett LW, Fletcher S, Wilton SD. Regulation of eukaryotic gene expression by the untranslated gene regions and other non-coding elements. Cell Mol Life Sci. 2012;69:3613–34. 10.1007/s00018-012-0990-9. 22538991 10.1007/s00018-012-0990-9 PMC3474909

[81] Quinn JJ, Chang HY. Unique features of long non-coding RNA biogenesis and function. Nat Rev Genet. 2016;17:47–62. 10.1038/nrg.2015.10. 26666209 10.1038/nrg.2015.10

[82] Doyle SR, Cotton JA. Genome-wide approaches to investigate anthelmintic resistance. Trends Parasitol. 2019;35:289–301. 10.1016/j.pt.2019.01.004. 30733094 10.1016/j.pt.2019.01.004

