# Supplementary figures and images for: Polymorphism-driven transcriptomic changes in anthelmintic metabolism pathways of Anisakis simplex s.s. L3 larvae
Source: Parasit Vectors. 2025 Dec 19;18:508. doi: 10.1186/s13071-025-07197-w (PMC12751375; doi:10.1186/s13071-025-07197-w)

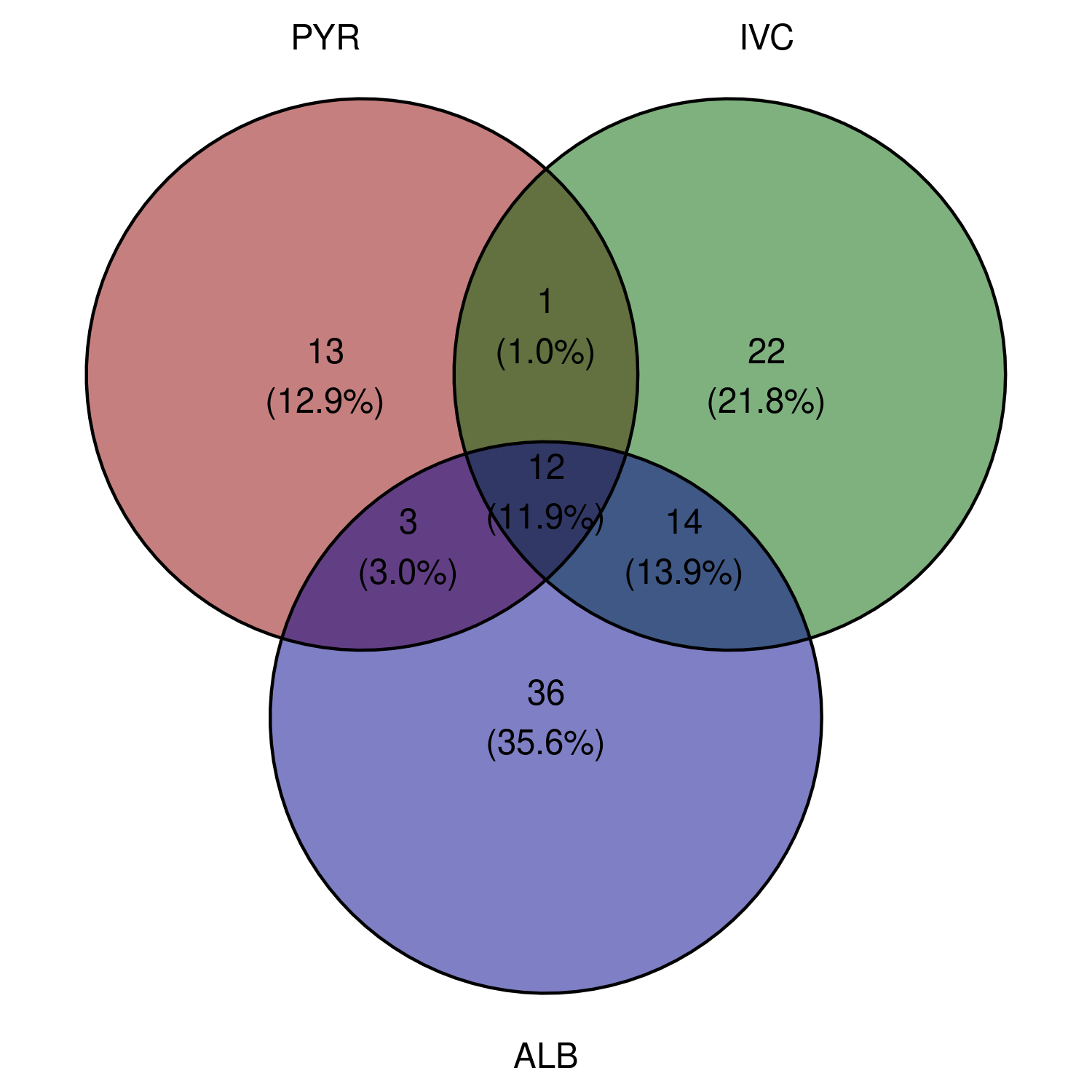

Supplement: Supplementary file 1 — Additional file 1: Figure S1. The Venn diagram illustrates the number of DEGs and the common DEGs in the ALB, IVC, PYRcolor comparisons. Figure S2. The Venn diagram illustrates the number of DELs and the common DELs in the ALB, IVC, PYRcolor comparisons. Figure S3. The results of correlations analysis between RT-PCR method and RNA-Seq. Selected DEGs were marked with different color for each treatment comparison. The Y-axis shows the log2 obtained by the RNA-seq method, while the X-axis shows the log2 of validated DEGs measured by the qPCR method. Details can be found in Additional file 2: Table S2. Figure S4. Relative expression levels of genes in response A. simplexto drug treatmentscompared to control, as determined by quantitative real-time PCR. mRNA expression of each genewas normalized to housekeeping gene expression and expressed relative to the control group. Data are presented as mean ± SEM from n = 4 samples. Statistical significance is indicated as p-values interpreted as follows: 0.0332, 0.0021, 0.0002, and <0.0001.^2045201,^1890101,^4133,^1857601,^1723901,^1578201,^1493901,^1486201,^1480801,^1418601,^1260001,^836301,^307801,^221901, ^74501,^22701. Figure S5. Volcano plot depicts the PSI levels forin the ALB experimental comparison. The X-axis represents the difference in PSI valuesfor each ASE, while the Y-axis displays the negative logarithmic FDR. A horizontal dotted line indicates the negative logarithmic value of the FDR cutoff, and two vertical lines represent the absolute ΔPSI value of 0.1. Colored points denote different types of significant DASes, and gray points represent non-significant DAS events. Figure S6. Volcano plot illustrating the PSI levels for significant DASes in the IVC experimental comparison. The X-axis displays the difference in PSI valuesfor each DASes, and the Y-axis shows the negative logarithmic FDR. A horizontal dotted line marks the negative logarithmic value of the FDR cutoff, while two vertical lines indicate the absolute [file 13071_2025_7197_MOESM1_ESM.zip › S1_Figure S1.png]

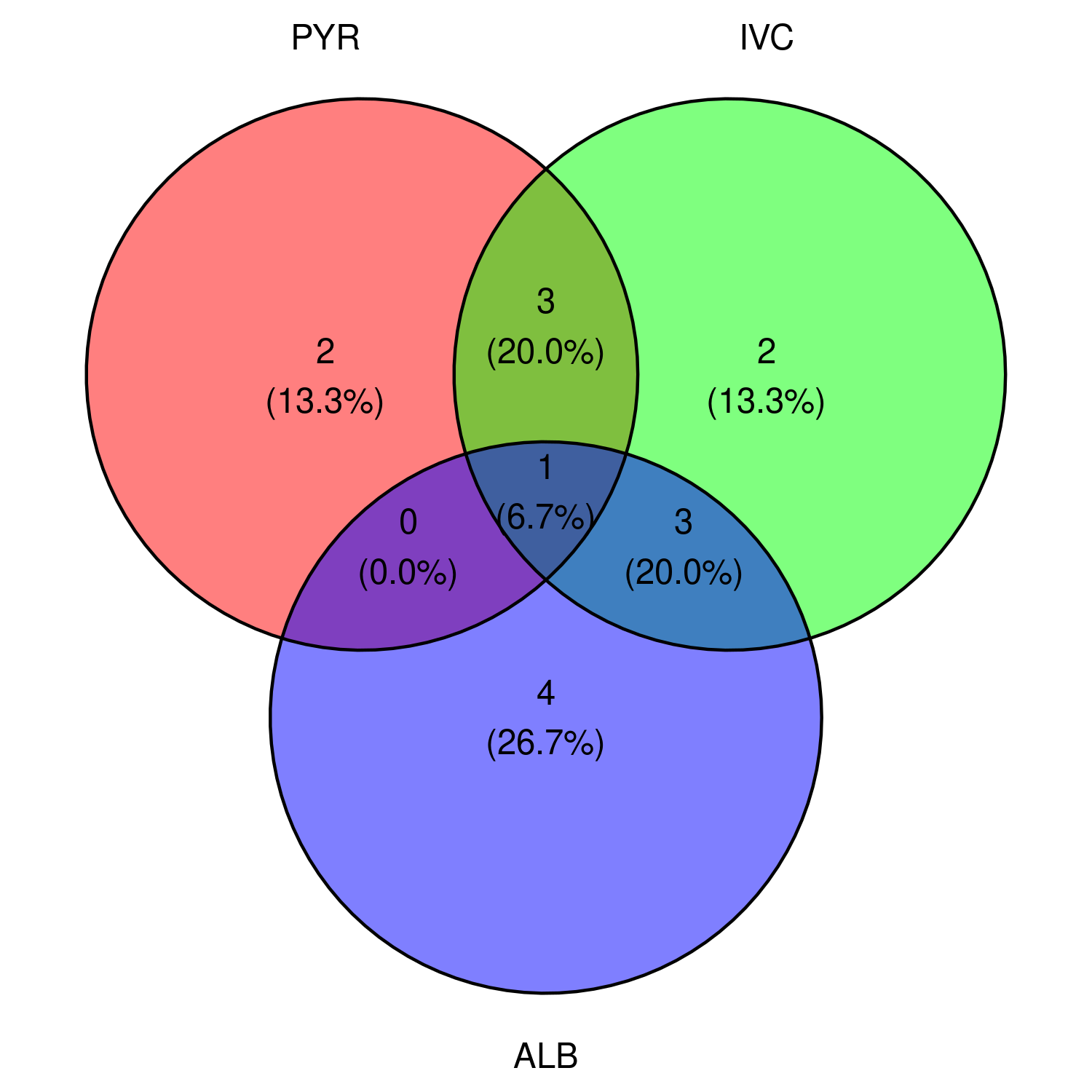

Supplement: Supplementary file 1 — Additional file 1: Figure S1. The Venn diagram illustrates the number of DEGs and the common DEGs in the ALB, IVC, PYRcolor comparisons. Figure S2. The Venn diagram illustrates the number of DELs and the common DELs in the ALB, IVC, PYRcolor comparisons. Figure S3. The results of correlations analysis between RT-PCR method and RNA-Seq. Selected DEGs were marked with different color for each treatment comparison. The Y-axis shows the log2 obtained by the RNA-seq method, while the X-axis shows the log2 of validated DEGs measured by the qPCR method. Details can be found in Additional file 2: Table S2. Figure S4. Relative expression levels of genes in response A. simplexto drug treatmentscompared to control, as determined by quantitative real-time PCR. mRNA expression of each genewas normalized to housekeeping gene expression and expressed relative to the control group. Data are presented as mean ± SEM from n = 4 samples. Statistical significance is indicated as p-values interpreted as follows: 0.0332, 0.0021, 0.0002, and <0.0001.^2045201,^1890101,^4133,^1857601,^1723901,^1578201,^1493901,^1486201,^1480801,^1418601,^1260001,^836301,^307801,^221901, ^74501,^22701. Figure S5. Volcano plot depicts the PSI levels forin the ALB experimental comparison. The X-axis represents the difference in PSI valuesfor each ASE, while the Y-axis displays the negative logarithmic FDR. A horizontal dotted line indicates the negative logarithmic value of the FDR cutoff, and two vertical lines represent the absolute ΔPSI value of 0.1. Colored points denote different types of significant DASes, and gray points represent non-significant DAS events. Figure S6. Volcano plot illustrating the PSI levels for significant DASes in the IVC experimental comparison. The X-axis displays the difference in PSI valuesfor each DASes, and the Y-axis shows the negative logarithmic FDR. A horizontal dotted line marks the negative logarithmic value of the FDR cutoff, while two vertical lines indicate the absolute [file 13071_2025_7197_MOESM1_ESM.zip › S1_ Figure S2.png]

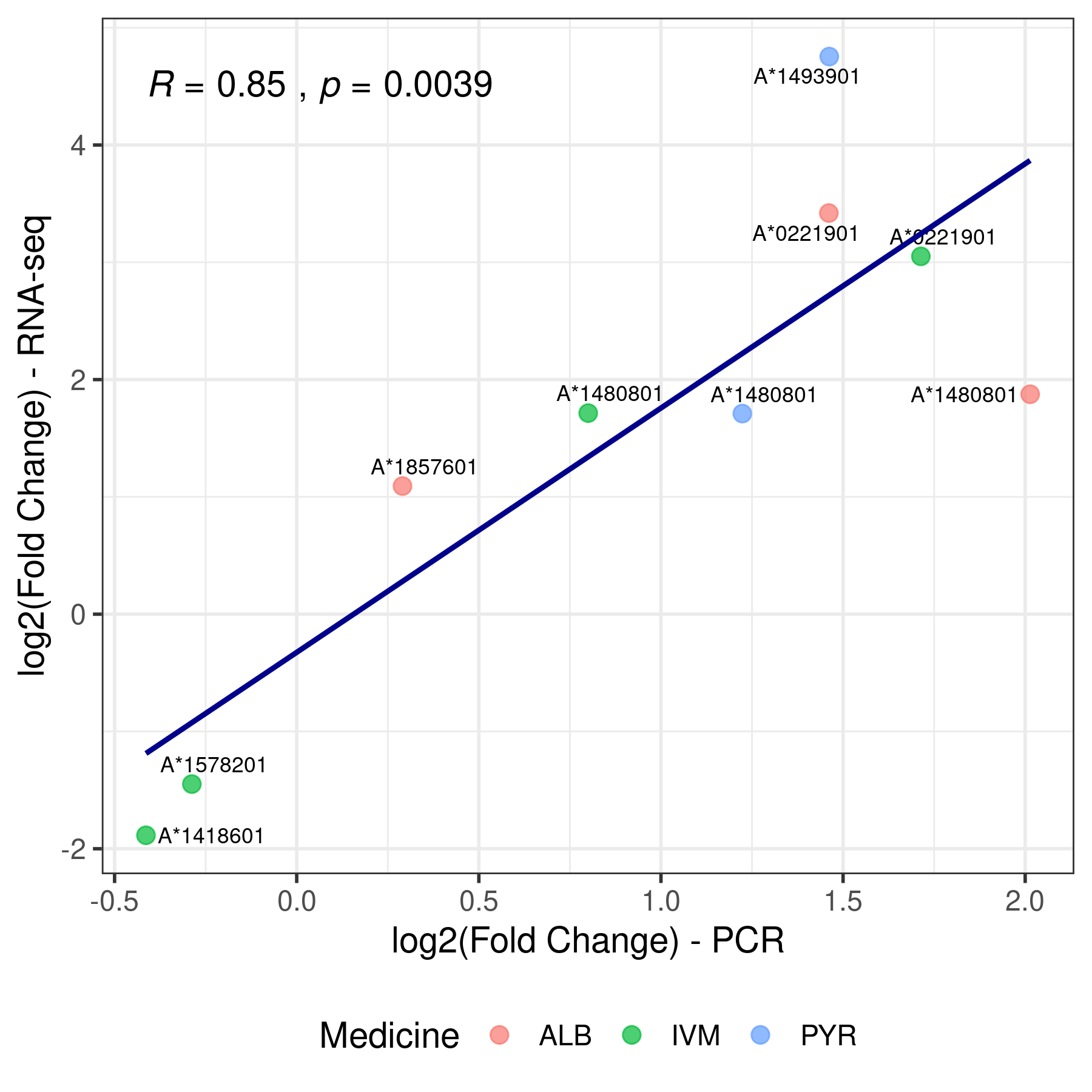

Supplement: Supplementary file 1 — Additional file 1: Figure S1. The Venn diagram illustrates the number of DEGs and the common DEGs in the ALB, IVC, PYRcolor comparisons. Figure S2. The Venn diagram illustrates the number of DELs and the common DELs in the ALB, IVC, PYRcolor comparisons. Figure S3. The results of correlations analysis between RT-PCR method and RNA-Seq. Selected DEGs were marked with different color for each treatment comparison. The Y-axis shows the log2 obtained by the RNA-seq method, while the X-axis shows the log2 of validated DEGs measured by the qPCR method. Details can be found in Additional file 2: Table S2. Figure S4. Relative expression levels of genes in response A. simplexto drug treatmentscompared to control, as determined by quantitative real-time PCR. mRNA expression of each genewas normalized to housekeeping gene expression and expressed relative to the control group. Data are presented as mean ± SEM from n = 4 samples. Statistical significance is indicated as p-values interpreted as follows: 0.0332, 0.0021, 0.0002, and <0.0001.^2045201,^1890101,^4133,^1857601,^1723901,^1578201,^1493901,^1486201,^1480801,^1418601,^1260001,^836301,^307801,^221901, ^74501,^22701. Figure S5. Volcano plot depicts the PSI levels forin the ALB experimental comparison. The X-axis represents the difference in PSI valuesfor each ASE, while the Y-axis displays the negative logarithmic FDR. A horizontal dotted line indicates the negative logarithmic value of the FDR cutoff, and two vertical lines represent the absolute ΔPSI value of 0.1. Colored points denote different types of significant DASes, and gray points represent non-significant DAS events. Figure S6. Volcano plot illustrating the PSI levels for significant DASes in the IVC experimental comparison. The X-axis displays the difference in PSI valuesfor each DASes, and the Y-axis shows the negative logarithmic FDR. A horizontal dotted line marks the negative logarithmic value of the FDR cutoff, while two vertical lines indicate the absolute [file 13071_2025_7197_MOESM1_ESM.zip › S1_ Figure S3.png]

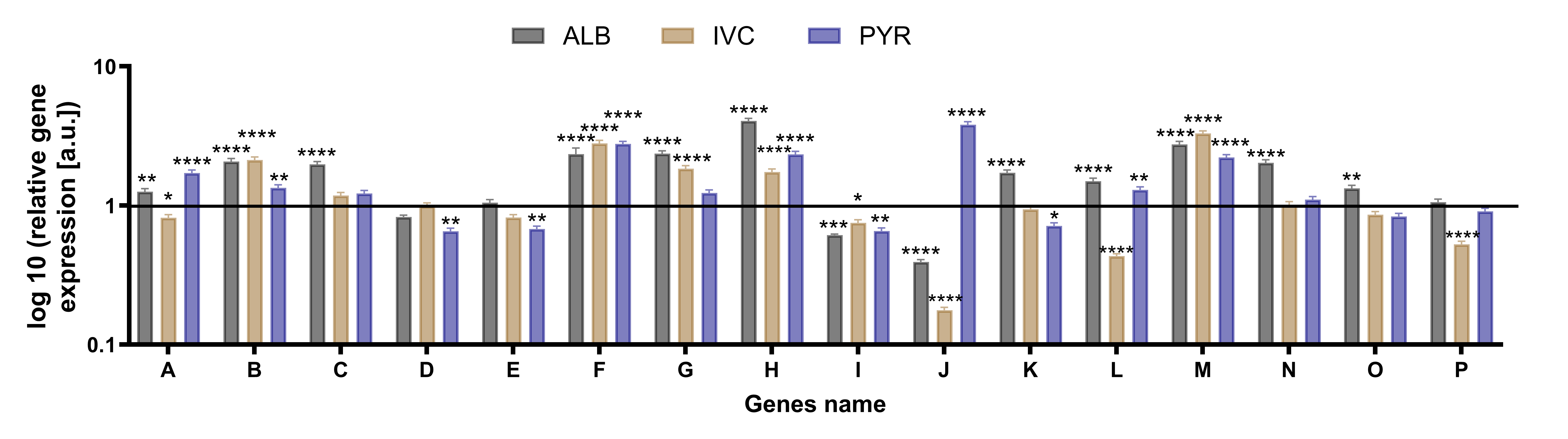

Supplement: Supplementary file 1 — Additional file 1: Figure S1. The Venn diagram illustrates the number of DEGs and the common DEGs in the ALB, IVC, PYRcolor comparisons. Figure S2. The Venn diagram illustrates the number of DELs and the common DELs in the ALB, IVC, PYRcolor comparisons. Figure S3. The results of correlations analysis between RT-PCR method and RNA-Seq. Selected DEGs were marked with different color for each treatment comparison. The Y-axis shows the log2 obtained by the RNA-seq method, while the X-axis shows the log2 of validated DEGs measured by the qPCR method. Details can be found in Additional file 2: Table S2. Figure S4. Relative expression levels of genes in response A. simplexto drug treatmentscompared to control, as determined by quantitative real-time PCR. mRNA expression of each genewas normalized to housekeeping gene expression and expressed relative to the control group. Data are presented as mean ± SEM from n = 4 samples. Statistical significance is indicated as p-values interpreted as follows: 0.0332, 0.0021, 0.0002, and <0.0001.^2045201,^1890101,^4133,^1857601,^1723901,^1578201,^1493901,^1486201,^1480801,^1418601,^1260001,^836301,^307801,^221901, ^74501,^22701. Figure S5. Volcano plot depicts the PSI levels forin the ALB experimental comparison. The X-axis represents the difference in PSI valuesfor each ASE, while the Y-axis displays the negative logarithmic FDR. A horizontal dotted line indicates the negative logarithmic value of the FDR cutoff, and two vertical lines represent the absolute ΔPSI value of 0.1. Colored points denote different types of significant DASes, and gray points represent non-significant DAS events. Figure S6. Volcano plot illustrating the PSI levels for significant DASes in the IVC experimental comparison. The X-axis displays the difference in PSI valuesfor each DASes, and the Y-axis shows the negative logarithmic FDR. A horizontal dotted line marks the negative logarithmic value of the FDR cutoff, while two vertical lines indicate the absolute [file 13071_2025_7197_MOESM1_ESM.zip › S1_ Figure S4.tif]

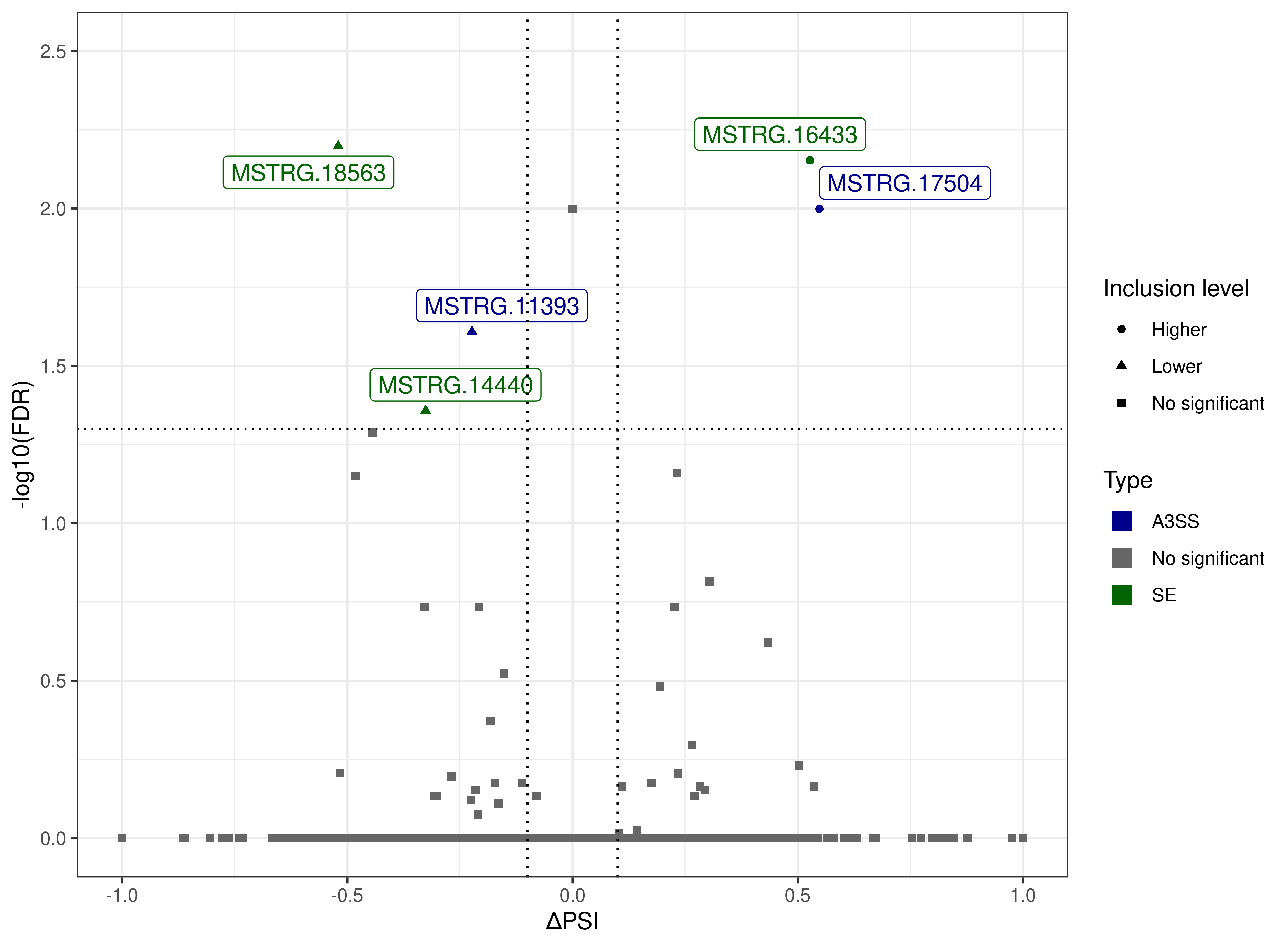

Supplement: Supplementary file 1 — Additional file 1: Figure S1. The Venn diagram illustrates the number of DEGs and the common DEGs in the ALB, IVC, PYRcolor comparisons. Figure S2. The Venn diagram illustrates the number of DELs and the common DELs in the ALB, IVC, PYRcolor comparisons. Figure S3. The results of correlations analysis between RT-PCR method and RNA-Seq. Selected DEGs were marked with different color for each treatment comparison. The Y-axis shows the log2 obtained by the RNA-seq method, while the X-axis shows the log2 of validated DEGs measured by the qPCR method. Details can be found in Additional file 2: Table S2. Figure S4. Relative expression levels of genes in response A. simplexto drug treatmentscompared to control, as determined by quantitative real-time PCR. mRNA expression of each genewas normalized to housekeeping gene expression and expressed relative to the control group. Data are presented as mean ± SEM from n = 4 samples. Statistical significance is indicated as p-values interpreted as follows: 0.0332, 0.0021, 0.0002, and <0.0001.^2045201,^1890101,^4133,^1857601,^1723901,^1578201,^1493901,^1486201,^1480801,^1418601,^1260001,^836301,^307801,^221901, ^74501,^22701. Figure S5. Volcano plot depicts the PSI levels forin the ALB experimental comparison. The X-axis represents the difference in PSI valuesfor each ASE, while the Y-axis displays the negative logarithmic FDR. A horizontal dotted line indicates the negative logarithmic value of the FDR cutoff, and two vertical lines represent the absolute ΔPSI value of 0.1. Colored points denote different types of significant DASes, and gray points represent non-significant DAS events. Figure S6. Volcano plot illustrating the PSI levels for significant DASes in the IVC experimental comparison. The X-axis displays the difference in PSI valuesfor each DASes, and the Y-axis shows the negative logarithmic FDR. A horizontal dotted line marks the negative logarithmic value of the FDR cutoff, while two vertical lines indicate the absolute [file 13071_2025_7197_MOESM1_ESM.zip › S1_ Figure S5.png]

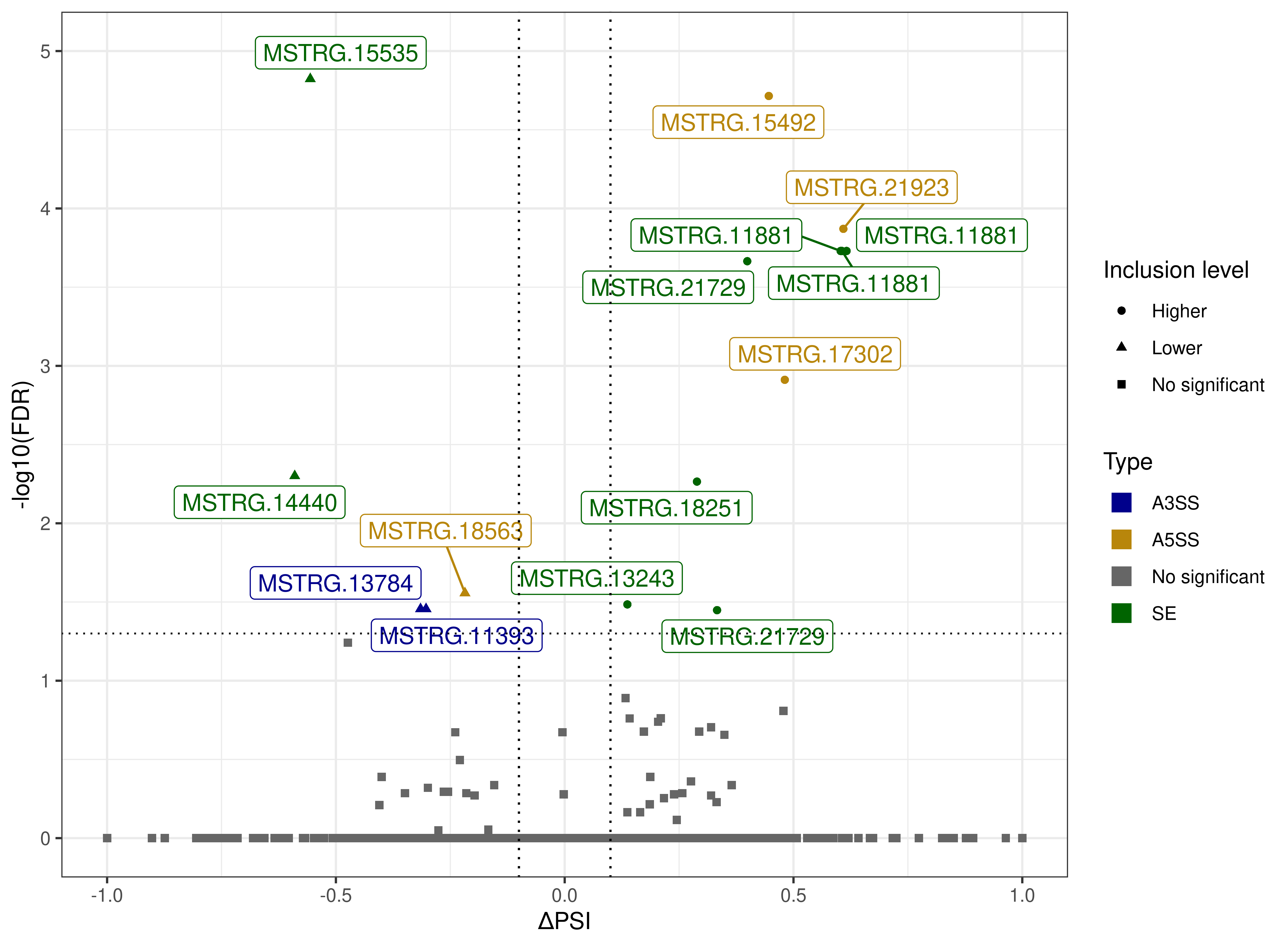

Supplement: Supplementary file 1 — Additional file 1: Figure S1. The Venn diagram illustrates the number of DEGs and the common DEGs in the ALB, IVC, PYRcolor comparisons. Figure S2. The Venn diagram illustrates the number of DELs and the common DELs in the ALB, IVC, PYRcolor comparisons. Figure S3. The results of correlations analysis between RT-PCR method and RNA-Seq. Selected DEGs were marked with different color for each treatment comparison. The Y-axis shows the log2 obtained by the RNA-seq method, while the X-axis shows the log2 of validated DEGs measured by the qPCR method. Details can be found in Additional file 2: Table S2. Figure S4. Relative expression levels of genes in response A. simplexto drug treatmentscompared to control, as determined by quantitative real-time PCR. mRNA expression of each genewas normalized to housekeeping gene expression and expressed relative to the control group. Data are presented as mean ± SEM from n = 4 samples. Statistical significance is indicated as p-values interpreted as follows: 0.0332, 0.0021, 0.0002, and <0.0001.^2045201,^1890101,^4133,^1857601,^1723901,^1578201,^1493901,^1486201,^1480801,^1418601,^1260001,^836301,^307801,^221901, ^74501,^22701. Figure S5. Volcano plot depicts the PSI levels forin the ALB experimental comparison. The X-axis represents the difference in PSI valuesfor each ASE, while the Y-axis displays the negative logarithmic FDR. A horizontal dotted line indicates the negative logarithmic value of the FDR cutoff, and two vertical lines represent the absolute ΔPSI value of 0.1. Colored points denote different types of significant DASes, and gray points represent non-significant DAS events. Figure S6. Volcano plot illustrating the PSI levels for significant DASes in the IVC experimental comparison. The X-axis displays the difference in PSI valuesfor each DASes, and the Y-axis shows the negative logarithmic FDR. A horizontal dotted line marks the negative logarithmic value of the FDR cutoff, while two vertical lines indicate the absolute [file 13071_2025_7197_MOESM1_ESM.zip › S1_ Figure S6.png]

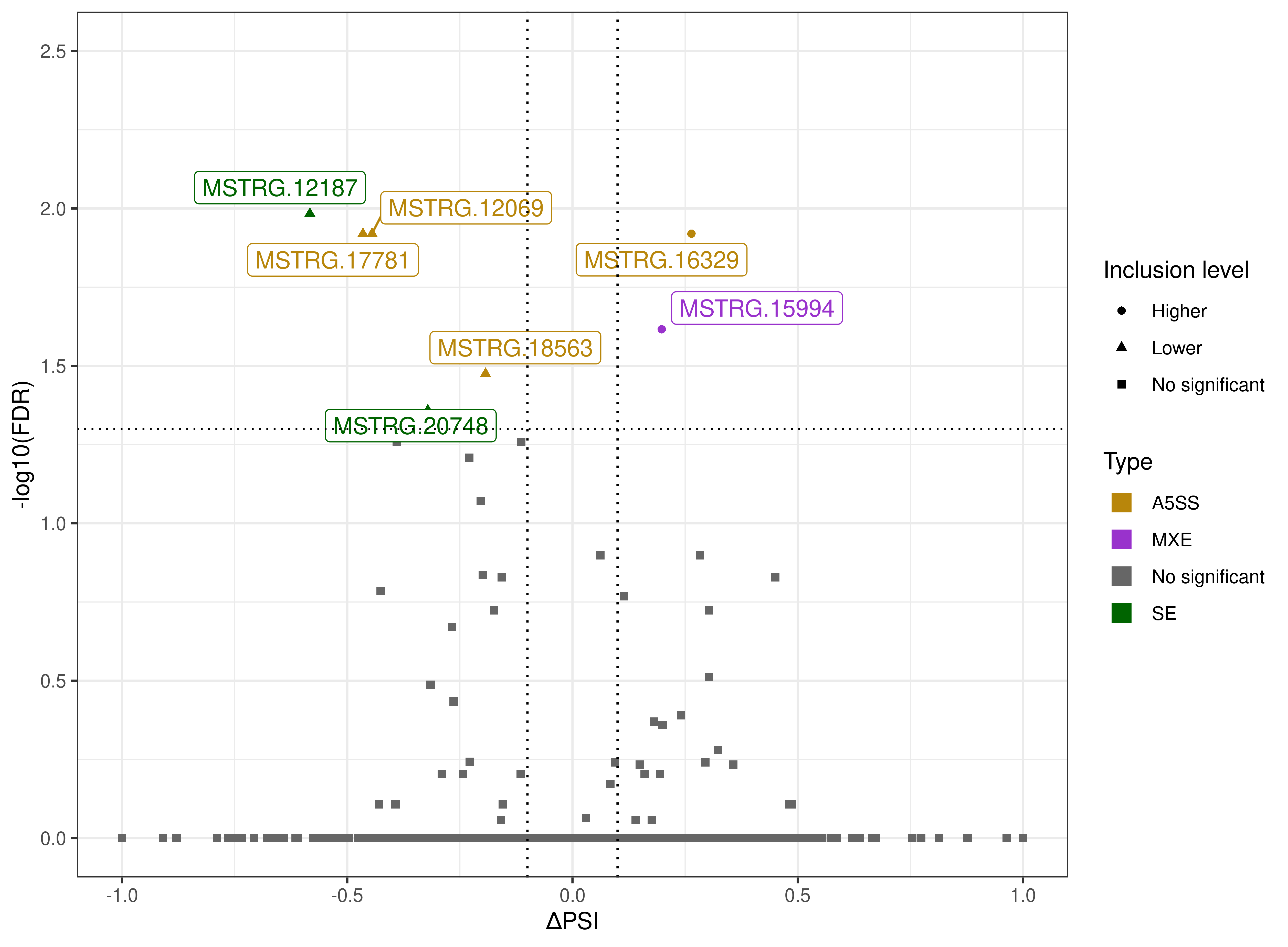

Supplement: Supplementary file 1 — Additional file 1: Figure S1. The Venn diagram illustrates the number of DEGs and the common DEGs in the ALB, IVC, PYRcolor comparisons. Figure S2. The Venn diagram illustrates the number of DELs and the common DELs in the ALB, IVC, PYRcolor comparisons. Figure S3. The results of correlations analysis between RT-PCR method and RNA-Seq. Selected DEGs were marked with different color for each treatment comparison. The Y-axis shows the log2 obtained by the RNA-seq method, while the X-axis shows the log2 of validated DEGs measured by the qPCR method. Details can be found in Additional file 2: Table S2. Figure S4. Relative expression levels of genes in response A. simplexto drug treatmentscompared to control, as determined by quantitative real-time PCR. mRNA expression of each genewas normalized to housekeeping gene expression and expressed relative to the control group. Data are presented as mean ± SEM from n = 4 samples. Statistical significance is indicated as p-values interpreted as follows: 0.0332, 0.0021, 0.0002, and <0.0001.^2045201,^1890101,^4133,^1857601,^1723901,^1578201,^1493901,^1486201,^1480801,^1418601,^1260001,^836301,^307801,^221901, ^74501,^22701. Figure S5. Volcano plot depicts the PSI levels forin the ALB experimental comparison. The X-axis represents the difference in PSI valuesfor each ASE, while the Y-axis displays the negative logarithmic FDR. A horizontal dotted line indicates the negative logarithmic value of the FDR cutoff, and two vertical lines represent the absolute ΔPSI value of 0.1. Colored points denote different types of significant DASes, and gray points represent non-significant DAS events. Figure S6. Volcano plot illustrating the PSI levels for significant DASes in the IVC experimental comparison. The X-axis displays the difference in PSI valuesfor each DASes, and the Y-axis shows the negative logarithmic FDR. A horizontal dotted line marks the negative logarithmic value of the FDR cutoff, while two vertical lines indicate the absolute [file 13071_2025_7197_MOESM1_ESM.zip › S1_Figure S7.png]
